# Cellular reagents for diagnostics and synthetic biology

**DOI:** 10.1371/journal.pone.0201681

**Published:** 2018-08-15

**Authors:** Sanchita Bhadra, Arti Pothukuchy, Raghav Shroff, Austin W. Cole, Michelle Byrom, Jared W. Ellefson, Jimmy D. Gollihar, Andrew D. Ellington

**Affiliations:** Department of Molecular Biosciences, College of Natural Sciences, The University of Texas at Austin, Austin, TX, United States of America; University of Helsinki, FINLAND

## Abstract

We have found that the overproduction of enzymes in bacteria followed by their lyophilization leads to 'cellular reagents' that can be directly used to carry out numerous molecular biology reactions. We demonstrate the use of cellular reagents in a variety of molecular diagnostics, such as TaqMan qPCR with no diminution in sensitivity, and in synthetic biology cornerstones such as the Gibson assembly of DNA fragments, where new plasmids can be constructed solely based on adding cellular reagents. Cellular reagents have significantly reduced complexity and cost of production, storage and implementation, features that should facilitate accessibility and use in resource-poor conditions.

## Introduction

Most molecular biology techniques commonly used in research, biotechnology, healthcare, and education rely heavily on purified functional protein reagents [1, 2]. For instance, nucleic acid amplification [3, 4] and editing [5]–cornerstones of molecular diagnostics and synthetic biology [6, 7]–typically depend on the activities of purified DNA and RNA polymerases, nucleases, and ligases. However, purification of these protein reagents requires substantial investment of time, expertise, equipment and infrastructure [8, 9], which at this point is primarily performed at the industrial scale. For instance, large batches (hundreds of milliliters to liters) of protein-expressing bacterial cultures need to be cultivated and subsequently processed using a complex set of procedures to lyse the bacteria and separate the proteins of interest from unwanted bacterial and extraction buffer contents [10, 11]. To facilitate these pipelines for production, proteins often must be modified with tags for chromatographic separation that are then removed following processing, adding additional steps and complexity to the purification procedure [12–14]. Furthermore, most desired proteins need to be maintained in a constant cold chain (4°C to -80°C), which not only raises the infrastructure cost for purification and storage, but also creates requirements for shipping and storage at points of use.

As a result, the affordability and accessibility of protein reagents can be significantly limited, especially in resource poor or remote settings [15, 16]. Conversely, simplification of the production, transportation, and storage of these enzymes and proteins would potentially reduce the cost, time, expertise, and infrastructure needed for application and thereby increase accessibility. Simplified methods for production and use of protein reagents might also encourage the development of local production at a less-than-industrial scale.

To enhance affordability and application of molecular biology reagents worldwide we have developed methodologies that simplify reagent production by eliminating protein purification. Instead, we have developed standardized protocols that use lyophilized bacteria as cellular packets of reagents (“cellular reagents”). These cellular reagents not only perform extremely well compared to their purified counterparts, but also are stable for long periods at ambient temperatures. In addition, most standard operating procedures for molecular biology are minimally perturbed–the pure protein reagent can be simply replaced by an optimal amount of the corresponding rehydrated, lyophilized cellular reagent.

To prove the general feasibility of our approach we have developed several cellular reagents for multiple molecular biology and diagnostics applications. These include DNA polymerases, such as KlenTaq [17], Taq [18], Bst-LF [19], Phusion [20, 21], and RTX, an engineered thermostable reverse transcriptase [22]. The cellular reagents perform on par with purified reagents in analytical procedures such as qPCR, reverse transcription qPCR, endpoint PCR analyzed by agarose gel electrophoresis, and loop-mediated isothermal amplification (LAMP) with fluorogenic strand displacement (OSD) probes [23]. Amplification efficiency, detection limits, and time to result were comparable to the same reactions performed with pure enzymes. Finally, in order to begin to transform how synthetic biology might be carried out in resource poor settings, we also use cellular reagents to demonstrate the synthesis of plasmids by Gibson assembly [24].

## Materials and methods

### Chemicals and reagents

All chemicals were of analytical grade and were purchased from Sigma-Aldrich (St. Louis, MO, U.S.A.) unless otherwise indicated. Bacterial growth media were purchased from Thermo Fisher Scientific (Waltham, MA). Bacterial strains and all pure enzymes and related buffers were purchased from New England Biolabs (NEB, Ipswich, MA) unless otherwise indicated. KlenTaq1 was purchased from DNA Polymerase Technologies (St. Louis, MO). All oligonucleotides and gene blocks were obtained from Integrated DNA Technologies (IDT, Coralville, IA, U.S.A.). Oligonucleotide and gene block sequences are summarized in **S1 Table**.

### Plasmids and cloning

PCR amplification of sequences for subsequent cloning was performed using Phusion DNA polymerase. Standard Gibson assembly techniques were used for all cloning unless otherwise noted. Coding sequences for shuffle-optimized KlenTaq DNA polymerase [25], Bst LF DNA polymerase [25], Taq DNA ligase (UniProtKB—B7A6G7), T5 Exonuclease (UniProtKB—P06229), MMLV reverse transcriptase (UniProtKB—P03355), Taq DNA polymerase [26], Phusion DNA polymerase [20, 21] and RTX thermostable reverse transcriptase [22] were cloned into pATetO 6xHis plasmid. This is an in-house designed plasmid based on the pASK-IBA37plus vector (IBA GmbH) from which the multiple cloning site, and Rop gene have been removed to improve plasmid copy number [25]. The plasmid also features a modified pAtetO promoter with a single point mutation to make it unidirectional. All enzyme coding sequences introduced into this vector were placed immediately downstream of the Factor X cleavage site. For some experiments, the coding sequences for wildtype and exonuclease deficient versions of RTX were cloned downstream of the T7 promoter in the pET21 vector (Sigma-Aldrich) [22]. Assembled plasmids were transformed into chemically competent Top10 *E*. *coli* and verified by Sanger sequencing at the Institute of Cellular and Molecular Biology Core DNA Sequencing Facility.

### Production of lyophilized cellular reagents

Top10, BL21 and BL21 DE3 strains of *E*. *coli* were used to prepare lyophilized cellular reagents. Chemically competent BL21 and BL21 DE3 bacteria were freshly transformed with pATetO and pET21 constructs, respectively, prior to each instance of cellular reagent preparation. Top10 strains transformed with pATetO constructs and stored as glycerol stocks at -80°C were used to inoculate fresh cultures for cellular reagent preparation. Overnight 3 ml cultures of transformed bacterial strains were grown in 2X YT broth containing 100 μg/ml ampicillin. Subsequently, 50 ml sub-cultures at 1:200 dilution, unless otherwise specified, were initiated in Superior Broth^TM^ (Athena Environmental Sciences, Inc., Baltimore, MD, USA) containing 100 μg/ml ampicillin. Sub-cultures were incubated in 250 ml conical flasks at 37°C and constant 225 rpm agitation. Bacterial growth was monitored by measuring absorbance of 600 nm wavelength light.

Protein production was initiated by inducing transcription from the pATetO and the pT7 promoters by adding 200ng/ml anhydrotetracycline (aTC) or 1 mM isopropyl β-D-1-thiogalactopyranoside (IPTG) to logarithm phase (typical A_600_ = 0.4 to 0.7) cultures. The pATetO promoter was induced for 3h at 37°C, unless otherwise indicated. The pT7 promoter was induced for 18 h at 18°C.

After induction, bacteria were collected by centrifugation followed by washing once in cold 1X PBS (137 mM NaCl, 2.7 mM KCl, 4.3 mM Na_2_HPO_4_, 1.47 mM KH_2_PO_4_, pH 7.4). The bacterial pellets were resuspended in cold 1X PBS at a density of A_600_ = 3.5 to 6.5. Some 2x10^8^ aTC-induced bacteria and 2x10^7^ IPTG-induced bacteria (estimated from the A_600_ value using the relation 0.5 optical density = 5x10^8^ bacteria/ml) were aliquoted into individual 0.2 ml PCR tubes and frozen at -80°C overnight prior to lyophilization for 3 h at 197 mTorr and -108°C using the automated settings in a VirTis Benchtop Pro lyophilizer (SP Scientific, Warminster, PA, USA). Lyophilized cellular reagents were stored with desiccant at room temperature, 37°C, or 42°C until use.

### Purification of RTX reverse transcriptase

RTX Exo- polymerase was expressed and purified in house following the protocol of Ellefson *et al* (2016) [22]. Briefly, BL21 DE3 bacteria harboring the pET21-RTX Exo- polymerase containing plasmid was grown overnight in Superior Broth^TM^ at 37°C. Cells were then diluted 1:200, and protein production was induced with 1 mM IPTG during mid-log phase at 18°C for 16–18 hrs. Harvested cells were flash-frozen and lysed by sonication in 10 mM phosphate, 100 mM NaCl, 0.1 mM EDTA, 1 mM DTT, 10% glycerol, pH 7 buffer containing protease inhibitor (Sigma-Aldrich). Cell lysate was then centrifuged at 40,000g for 45 min at 4°C. Cleared cell lysates were heated at 85°C for 25 min, cooled on ice for 20 min, and spun again at 20,000g for 15 min. Supernatant obtained after centrifugation was filtered using 0.2 μm filters. The filtrate was then passed over an equilibrated heparin column (GE Life Sciences, Pittsburgh, PA, USA), and eluted along a sodium chloride gradient. Polymerase fractions were collected and dialyzed into Buffer A [22]. Enzymes were further purified using an SP column (GE Life Sciences) and again eluted along a salt gradient. Pooled fractions were then applied to a Sephadex 16/60 size exclusion column (GE Life Sciences), concentrated, and dialyzed into storage buffer (50 mM Tris-HCl, 50 mM KCl, 0.1 mM EDTA, 1 mM DTT, 0.1% Nonidet P40, 0.1% Tween-20, 50% glycerol, pH 8.0). Purified RTX Exo- polymerase was quantified by Pierce BCA protein assay kit (Thermo Fisher Scientific).

### Overlap extension assay using Taq DNA polymerase

Taq DNA polymerase-expressing Top10 *E*. *coli* cells were cultured and processed as cellular reagents as described above. Prior to freeze drying, an aliquot containing 2 x 10^8^ of these freshly cultured cells was resuspended in 30 μL 1X PBS and centrifuged for 1 min at 13,000 rpm. The resulting supernatant was collected in a fresh tube while the cell pellet was resuspended in 30 μL water. Taq DNA polymerase activities were measured in 3 μL aliquots of the supernatant and in 2 x 10^7^ prepared cells (contained in 3 μL aliquots) using overlap extension assays executed as follows. Forty seven microliter reactions containing 2 μM each of overlapping oligonucleotides OE.FWD and OE.REV, 1X Thermopol buffer (NEB) (20 mM Tris-HCl, 10 mM (NH4)_2_SO_4_, 10 mM KCl, 2 mM MgSO_4_, 0.1% Triton®-X-100, pH 8.8), and 0.2 mM deoxyribonucleotides (dNTPs) were assembled and heated to 95°C for 1 min followed by cooling on ice for 2 min. Then, 3 μL supernatant or 3 μL cells (2x10^7^) were added to the reactions, which were then incubated for 1 h at 37°C, 42°C, 65°C, or 75°C. Negative control reactions were performed in the same manner with the exception that water was used instead of OE.FWD and OE.REV oligonucleotide templates. A second aliquot of 2 x 10^8^ cells was first frozen in 1X PBS at– 80°C prior to testing for Taq DNA polymerase activity in cells and supernatant as described above. A third aliquot of 2 x 10^8^ cells was frozen in 1X PBS and then lyophilized prior to rehydration with 30 μL water and testing for Taq DNA polymerase activity in oligonucleotide extension assays. Supernatant could not be separated from this rehydrated sample under the centrifugation conditions described above. All oligonucleotide extension products were analyzed by ethidium bromide agarose gel electrophoresis.

### Endpoint PCR using fresh broth culture of cellular reagents

BL21 DE3 bacteria transformed with RTX Exo- polymerase expression plasmid were grown to logarithm phase in Superior Broth^TM^ and induced with 1 mM IPTG as described in Section 2.3. One milliliter culture of induced cells was centrifuged at 16,000g for 1 min. Supernatant was removed and bacteria were resuspended in 1 ml PBS. 1 μl of this neat or 1:10 diluted bacterial suspension was added to a 20 μl PCR reaction containing 10 ng (1 x 10^8^ copies) of *Chlamydia trachomatis* 16s rDNA templates in 1X PCR proof reading assay buffer (60 mM Tris-HCl (pH8.4), 25 mM (NH4)_2_SO_4_, 10 mM KCl and 1 mM MgSO_4_) supplemented with 0.5 mM dNTPs and 400 nM each of forward (CT.F) and reverse (CT.R) primers. In positive control PCR reactions, RTX Exo- expressing bacterial suspension was replaced with 1 μl of purified RTX Exo- polymerase (0.2 mg/ml stock) or with commercially available KOD DNA polymerase (Sigma Aldrich, St. Louis, MO). Reactions were incubated at 95°C for 5 min followed by 25 cycles of 20 sec at 95°C, 20 sec at 55°C, and 20 sec at 68°C. PCR products were analyzed by electrophoresis through a 1.5% agarose gel prepared in 1X TAE Buffer (22 mM Tris, 180 mM-borate, 5 mM EDTA pH 8.3). Some 0.5 μg/ml of ethidium bromide was included in the gel to visualize the DNA bands under UV light. After running the gel at 80 Volts for 30 min, bands were visualized using ChemiDoc Imager (Bio-Rad).

### Endpoint PCR using Taq DNA polymerase lyophilized cellular reagents

Endpoint PCR reactions were assembled in 50 μL volumes containing zero or 10 ng of a pCR2.1-FluB plasmid template along with a final concentration of 500 nM each of pCR.FWD and pCR.REV primers. Amplification was performed in 1X Thermopol buffer (NEB) containing 0.2 mM dNTPs and 3 μL (2 x 10^7^ cells) of Taq DNA polymerase cellular reagent rehydrated in 30 μL water immediately prior to use. Following an initial 10 min incubation at 95°C, the reactions were subjected to 30 thermal cycles of 30 sec at 95°C, 30 sec at 55°C, and 1 min at 72°C. Ten microliters of the resultant PCR products were analyzed by agarose gel electrophoresis.

### Real-time LAMP-OSD

LAMP-OSD reaction mixtures were prepared in 25 μl volume containing indicated amounts of human glyceraldehyde-3-phosphate dehydrogenase (*gapd*) DNA templates along with a final concentration of 1.6 μM each of BIP and FIP primers, 0.4 μM each of B3 and F3 primers, and 0.8 μM of the loop primer. Amplification was performed in 1X isothermal buffer (NEB) (20 mM Tris-HCl, 10 mM (NH4)_2_SO_4_, 10 mM KCl, 2 mM MgSO_4_, 0.1% Triton X-100, pH 8.8) containing 0.8 M betaine, 0.8 mM dNTPs, 2 mM additional MgSO_4_, 16 units of pure Bst 2.0 DNA polymerase, and 100 nM of OSD reporter. Reporters were prepared for use in LAMP assays by annealing 100 nM fluorophore-labeled OSD strands with a 5-fold excess of the quencher-labeled OSD strands by incubation at 95°C for 1 min followed by cooling at the rate of 0.1°C/sec to 25°C). In some LAMP-OSD assays commercial Bst 2.0 was substituted either with 16 units of commercial Bst-LF DNA polymerase or with 3 μl (2 x 10^7^ cells) of Bst-LF expressing lyophilized BL21 cellular reagents (rehydrated prior to use in 30 μl water). For real-time signal measurement these LAMP-OSD reactions were transferred into a 96-well PCR plate, which was incubated in a LightCycler 96 real-time PCR machine (Roche, NC, U.S.A.) maintained at 65°C for 90 min. Fluorescence signals were recorded every 3 min and analyzed using the LightCycler 96 software.

### Quantitative PCR

KlenTaq DNA polymerase qPCR reactions were prepared in 25 μl volume containing indicated amounts of *Chlamydia trachomatis* 16S DNA templates along with a final concentration of 0.4 μM each of forward (CT.F) and reverse (CT.R) primers. Amplification was performed in 1X KlenTaq1 buffer (DNA Polymerase Technology) (50mM Tris-Cl pH 9.2, 16 mM ammonium sulfate, 0.05% Brij 58, and 3.5 mM magnesium chloride) containing 0.4 mM dNTPs, 0.2 μl of pure KlenTaq1 DNA polymerase, and 1X EvaGreen intercalating dye (Biotium, Freemont, CA). In some qPCR assays, commercial KlenTaq1 was substituted with 3 μl (2 x 10^7^ cells) of KlenTaq expressing lyophilized BL21 cellular reagents (rehydrated prior to use in 30 μl water). For real-time signal measurement these qPCR reactions were transferred into a LightCycler 96 real-time PCR machine and subjected to 10 min at 95°C followed by 45 cycles of 10 sec at 95°C (denaturation), 30 sec at 55°C (annealing) and 30 sec at 72°C (extension). Fluorescence signals were recorded during the extension step in each cycle. Following qPCR amplicon melting curve analysis was performed. All data were analyzed using the LightCycler 96 software.

Taq DNA polymerase TaqMan qPCR reactions were prepared in 25 μl volume containing indicated amounts of Zika virus NS5 DNA templates along with a final concentration of 0.32 μM each of forward (Zika-4481_F) and reverse (Zika-4552c) primers [27]. Amplification was performed in 1X Thermopol buffer (NEB) containing 0.4 mM dNTPs, 2.5 units of Taq DNA polymerase, and 80 nM TaqMan probe (Zika-4507c-FAM) [27]. In some assays commercial Taq DNA polymerase was substituted with 3 μl (2 x 10^7^ cells) of Taq DNA polymerase expressing lyophilized BL21 cellular reagents (rehydrated prior to use in 30 μL water). For real-time signal measurement these TaqMan qPCR reactions were transferred into a LightCycler 96 real-time PCR machine and subjected to 10 min at 95°C followed by 45 cycles of 15 sec at 95°C (denaturation) and 30 sec at 55°C (annealing and extension). Fluorescence signals were recorded during the annealing/extension step in each cycle. All data were analyzed using the LightCycler 96 software.

RTX reverse transcriptase qPCR reactions were prepared in 20 μl volume containing indicated amounts of *Chlamydia trachomatis* or Zika virus-derived DNA templates along with a final concentration of 200 nM each of forward and reverse primers (CT.F/R and Zika-255-F/Zika-256-R, respectively). Amplification was performed in 1X PCR proof reading assay buffer (60 mM Tris-HCl (pH8.4), 25 mM (NH4)_2_SO_4_, 10 mM KCl and 1 mM MgSO_4_) containing 0.5 mM dNTPs, 1.5 M Betaine, 1X EvaGreen dye and 80 ng of RTX Exo- polymerase. In some assays, purified RTX polymerase was substituted with 5μl (2X10^6^ cells) of lyophilized cellular reagent (rehydrated prior to use in 50 μl water). For real-time signal measurement these qPCR reactions were transferred into a LightCycler 96 real-time PCR machine and subjected to 5 min at 95°C followed by 45 cycles of PCR. *Chlamydia trachomatis* template was cycled through 20 sec at 95°C, 20 sec at 55°C and 20 sec at 68°C while Zika template was cycled through 30 sec at 95°C, 30 sec at 55°C and 30 sec at 68°C. Fluorescence signals were recorded during the extension (68°C) step in each cycle. All data were analyzed using the LightCycler 96 software.

### Two-step quantitative reverse transcription (RT) PCR

Indicated amounts of *in vitro* transcribed and polyacrylamide gel purified Zika virus NS5 RNA templates were mixed with 10 μM reverse (Zika-4552c) primers and 1 mM dNTP in a total volume of 10 μl. Primer template annealing was performed by incubating the solutions at 65°C for 5 min followed by 2 min on ice. Reverse transcription was initiated by adding a 10 μl solution containing 2X MMLV RT buffer (NEB) (100 mM Tris-HCl, 20 mM DTT, 150 mM KCl, 6 mM MgCl_2_, pH 8.3), 8 units of RNase inhibitor and 3 μl (2 x 10^7^ cells) of MMLV reverse transcriptase expressing lyophilized cellular reagents (rehydrated prior to use in 30 μl water). Following 1 h of reverse transcription at 42°C, 5 μl of the resulting cDNA-containing solution was analyzed by TaqMan qPCR using Taq DNA polymerase expressing lyophilized BL21 cellular reagents as described above.

### One-pot quantitative reverse transcription (RT) PCR using RTX polymerase

One-pot RT-qPCR reactions using pure or cellular RTX Exo- reagent and indicated copies of Zika virus-derived RNA templates were assembled using the same procedure as RTX qPCR described above but with the addition of 10 mM DTT. For real-time signal measurement these RT-qPCR reactions were transferred into a LightCycler 96 real-time PCR machine and subjected to 68°C for 30 min followed by 5 min at 95°C prior to 45 cycles of 30 sec at 95°C, 30 sec at 55°C and 30 sec at 68°C. Fluorescence signals were recorded during the extension (68°C) step in each cycle. All data were analyzed using the LightCycler 96 software.

### Cellular PCR (cPCR) using lyophilized Phusion cellular reagents

Bacteria containing target DNA sequences were grown overnight at 37°C and 250 rpm in 3 ml 2X YT broth containing the appropriate antibiotics for selective pressure. Following overnight growth, cultures with A_600_ of 5 to 6 were diluted 1:10 in sterile water. Two microliters of this diluted culture was added to cPCR reaction to initiate amplification. cPCR reactions were assembled in a total volume of 50 μl containing 1X HF buffer (NEB), 0.2 mM dNTP mix, 0.2 μM each of forward and reverse primers and 3 μl of Phusion cellular reagent (prepared by rehydrating 2x10^8^ lyophilized Top10 *E*. *coli* expressing Phusion DNA polymerase in 30 μl water). cPCR reactions were incubated at 95°C for 10 min followed by 30 cycles of 30 sec at 95°C, 30 sec at 60°C, and 5 min at 72°C. cPCR products were analyzed by agarose gel electrophoresis and used directly for Gibson assembly. A portion of cPCR products were subjected to agarose gel purification using Wizard SV gel purification kit (Promega, Madison, WI, USA) prior to use in Gibson assembly.

### Gibson assembly and transformation of chemically competent bacteria

Twenty microliters Gibson assembly reactions were assembled by mixing vectors and inserts in 1X Gibson assembly buffer (0.1 M Tris-HCl, pH 7.5, 0.01 M MgCl_2_, 0.2 mM dGTP, 0.2 mM dATP, 0.2 mM dTTP, 0.2 mM dCTP, 0.01 M DTT, 5% (w/v) PEG-8000, 1 mM NAD) supplemented with pure enzymes or with cellular reagents. Assemblies using pure enzymes contained 0.08 units of T5 exonuclease (NEB), 0.5 units of Phusion DNA polymerase (NEB) and 80 units of Taq DNA ligase (NEB). For Gibson assemblies using cellular reagents the pure enzymes were substituted with individual Top10 *E*. *coli* cellular reagents expressing Taq DNA Ligase, Taq DNA polymerase, and T5 exonuclease.

Lyophilized cellular reagents were prepared as follows for use in Gibson assemblies. 2x10^8^ Top10 lyophilized cellular reagents expressing Taq DNA polymerase, T5 exonuclease, or Taq DNA ligase were rehydrated using 30 μl of water. The rehydrated T5 exonuclease cellular reagent was diluted 1:100 in water followed by addition of 1.5 μl aliquot per Gibson reaction. Rehydrated Taq DNA polymerase and Taq DNA Ligase cellular reagents were incubated at 75°C for 10 min. Three microliters of the heat-treated cellular Taq DNA Ligase was directly added to cellular Gibson assemblies. Heat-treated Taq DNA polymerase cellular reagents were diluted 1:10 in water prior to addition of 1.5 μl aliquot per Gibson assembly.

Linearized vectors and inserts for Gibson assemblies were mixed in the following ratios: Two part assemblies of double stranded gene block (gBlock) inserts (IDT) and linearized vector (agarose gel purified PCR product) contained equimolar ratio of vector (30 ng) and gBlock (3 ng). Two part assemblies of cPCR-amplified vectors and inserts contained 2.5 μl each of unpurified or gel-purified vector and insert cPCR products. Three-part cPCR vector and insert assemblies contained 1.5 μl of cPCR vector fragment 1, 1.5 μl of cPCR vector fragment 2 and 2 μl of insert cPCR.

Negative controls for Gibson assemblies included vectors and inserts in 1X Gibson buffer without any pure enzymes or cellular reagents. All Gibson assemblies were incubated at 50°C for 1 h prior to transformation.

One microliter of each Gibson assembly was transformed directly into chemically-competent Top10 *E*. *coli* bacteria. Briefly, 50 μl home-made competent bacteria [28] were mixed with the Gibson assembly and incubated on ice for 15 min. Following a 30 sec heat-shock at 42°C and a 2 min incubation on ice bacteria were allowed to recover for 1 h in 250 μl SOC medium (0.5% Yeast Extract, 2% Tryptone, 10 mM NaCl, 2.5 mM KCl, 10 mM MgCl_2_, 10 mM MgSO_4_, 20 mM Glucose) at 37°C on a rotator. All bacteria were collected by centrifugation and plated on 2X YT agar plates containing appropriate antibiotic selection. Following 18 h at 37°C bacterial colonies were counted to determine efficiency of Gibson assembly. Representative colonies were verified by Sanger sequencing.

### Statistical analysis

A minimum of three biological replicates with duplicate or triplicate embedded technical replicates were performed. ANOVA testing was used to determine whether cellular reagents significantly affect the Cq value of a reaction. Two different ANOVA tests were performed. One for PCR like reactions and one for LAMP based reactions. For LAMP based reactions, cellular Bst-LF was compared to purified Bst-2.0. Effect sizes were determined using linear regression. The Cq response was modeled as a function of template copy number, the reaction type (e.g. RTX-qPCR, EVG-qPCR KlenTaq, etc.), and reagent type (purified or cellular). The effect size of cellular reagents on Cq value was determined using the “effects” package from the Comprehensive R Archive Network (CRAN) repository. Reactions with either zero template copies or no observed Cq values were omitted from analysis. All models were built using RStudio version 1.0.136. The data modeled, scripts, and a more detailed summary of the statistical analysis are included as supplements (**S1 Statistical Supplement**).

## Results

### Development of cellular reagents for PCR

To determine the feasibility of using bacteria that express heterologous proteins directly as reagents in molecular biology reactions we carried out PCR using fresh cultures of bacteria expressing RTX Exo- polymerase. We observed bands of the expected size (~ 200 bp), similar to a positive control that contained purified RTX Exo- polymerase or KOD DNA polymerase (**S1 Fig**). These results suggest that bacteria expressing a polymerase enzyme could indeed be directly used as reagents without requiring prior purification of the polymerase.

While the use of fresh cultures for promulgating reactions presents many opportunities, it can also prove cumbersome. Therefore, we attempted to show that cellular reagents could also be stored and used. Overexpressing bacteria were either stored at -80°C or lyophilized, prior to functional comparison with pure enzymes in qPCR reactions. Enzyme activities of cellular reagents versus corresponding pure enzymes were compared by measuring the Cq values (time to detection) for the same number of template copies. Since there are typically yield losses during protein purification, it is difficult to accurately measure the physical amount of enzyme present in cellular reagents. Therefore, we compared the enzyme activity of defined numbers of RTX Exo- bacterial cellular reagents with the activity of corresponding pure RTX enzymes used at an experimentally determined optima of 80 ng / reaction. Similar C_q_ values for target detection were obtained irrespective of whether 2 x 10^6^ lyophilized cells/reaction or 80 ng of purified polymerases were used (**S2 Fig**). Frozen cellular reagents or 2 x 10^5^ lyophilized cells/reaction yielded somewhat higher (less sensitive) C_q_ values (**S2 Fig**).

Given that conventional wisdom would suggest that the addition of a highly complex mixture of enzymes and metabolites (i.e., a cell, lyophilized or lysed) to a reaction might have resulted in significant background amplification, an examination of additional negative controls was especially important. While purified enzyme did not generate spurious amplicons, frozen or lyophilized bacteria expressing RTX Exo- polymerase generated a small amount of non-specific signal in the absence of templates. These non-specific signals, however, could be readily distinguished from specific amplicons by performing melt curve analysis. Lyophilized BL21 DE3 bacteria that do not harbor the RTX Exo- polymerase generated linear background curves without measurable Cq values when presented with 1 x 10^8^ templates. Since lyophilized cellular reagents demonstrated comparable qPCR performance as pure enzymes and were stable at room temperature storage for at least 80 days (**S3 Fig**) we chose to focus on the development of lyophilized cellular reagent toolkits for potential diagnostic and molecular biology applications. Lyophilized cellular reagents have the added advantage of biosafety; *E*. *coli* lyophilized without excipients (such as trehalose) do not retain viability (**S4 Fig**) [29, 30], and therefore are safer for distribution and use.

It was possible that cellular reagents might be limited to the RTX enzyme, which had been evolved via emulsion-based selections that involved whole bacteria, and was therefore pre-optimized to function in the presence of bacterial components. Moreover, it was unclear whether cellular reagents required bacterial lysis, such as by heat treatment [31] due to cycling through 95°C during PCR, to facilitate enzyme access, a step that might limit the development of mesophilic cellular reagents.

Therefore, we attempted to determine if other enzymes commonly used for PCR could be similarly repackaged as simpler ready-to-use cellular reagents. *E*. *coli* cells overexpressing Taq DNA polymerase were washed in PBS and assessed for enzyme activity in three different conditions: fresh cells, cells frozen at -80°C, or lyophilized cells. Cells were tested isothermally by single step overlap extension assays at four different temperatures– 37°C, 42°C, 65°C, and 75°C–to evaluate accessibility. The PBS supernatants leftover after pelleting fresh or frozen cells were also tested for polymerase activity. Rehydrated lyophilized bacteria could not be separated from supernatant under similar centrifugation conditions. Our results demonstrate that most of the Taq DNA polymerase activity is associated with bacterial cells (**S5 Fig**). Small amounts of activity evident in the supernatants is likely due to contaminating cells and/or cellular components that were not removed by centrifugation. Amplicon output of fresh and frozen cells was similar to that of pure Taq DNA polymerase only at temperatures ≥65°C, suggesting that these cells likely have intact cell walls that restrict enzyme accessibility and hence observable activity at more mesophilic temperatures. In contrast, the yield of amplicons generated by rehydrated lyophilized cellular reagents was similar to pure enzyme at all temperatures, suggesting unhindered access to the enzyme payload of cellular reagents. It seems osmotic shock may have the same effect as heat treatment for lyophilized cells.

Microscopic examination of Gram-stained rehydrated cellular reagents revealed that most *E*. *coli* cells lyophilized in 1X PBS were not dispersed but appeared hollow, unlike similarly stained fresh cells or cells lyophilized in water (**S6 Fig**). In contrast, upon heating for 5 min at 95°C rehydrated cells that had been lyophilized in 1X PBS were found to disintegrate while, most rehydrated cells that had been lyophilized in water retained their shape even after heat treatment. These observations suggests that *E*. *coli* freeze-dried in 1X PBS might have porous walls that allow intermingling of cellular and external reaction contents upon rehydration.

**Fig 1:**
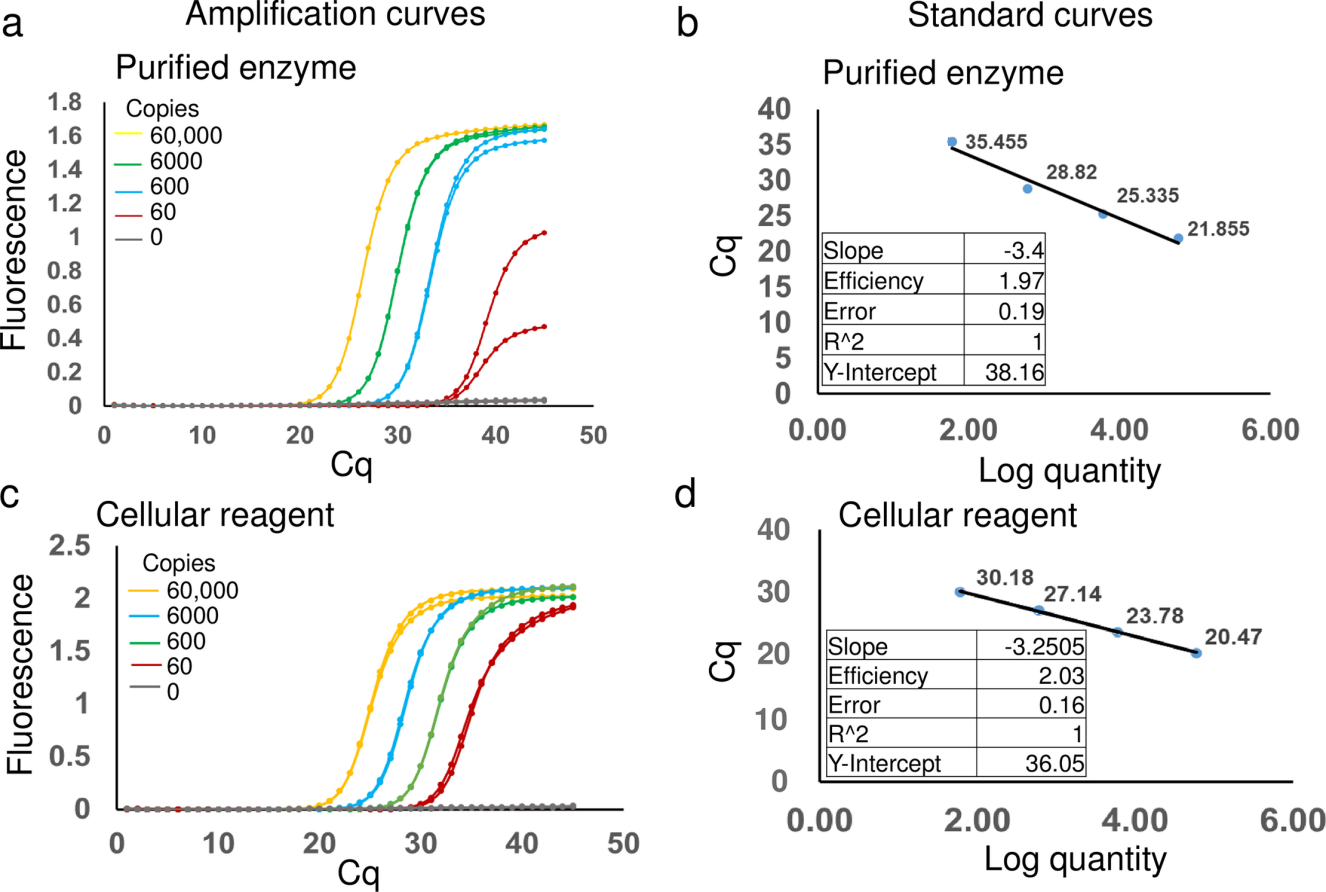
TaqMan qPCR analysis using lyophilized Taq DNA polymerase cellular reagents. Indicated copies of synthetic DNA templates derived from Zika virus genomic sequence were amplified using 2.5 units of pure commercial Taq DNA polymerase (panels a and b) or 2 x 10^7^ cells of rehydrated cellular reagents expressing Taq DNA polymerase (panels c and d). Amplification was assessed in real-time by measuring increase in TaqMan probe fluorescence over time. Representative amplification curves generated using the “Abs quant” analysis in the LightCycler 96 software are depicted in panels a and c. Amplification curve colors distinguish starting template copies–yellow: 60,000 template copies; green: 6000 template copies; blue: 600 template copies; red: 60 template copies; and gray: no template control. These curves depict the real-time kinetics of PCR amplification mediated by pure versus cellular reagents. The corresponding standard curve analyses performed using the “Abs quant” protocol in the LightCycler 96 software are depicted in panels b and d, respectively. Standard curve analyses data for comparing amplification efficiency, linearity, and error are tabulated as insets.

Enzymes other than RTX reverse transcriptase should be compatible with a cellular reagent format, especially since bacterial lyophilization in the presence of PBS is sufficient for enzyme access and additional bacterial lysis procedures are not required. In support of this hypothesis, Taq DNA polymerase cellular reagents also do not require cold storage; at the time of this publication these reagents remained functional even after 3 weeks at temperatures as high as 42°C (**S7 Fig**). Indeed, Taq DNA polymerase cellular reagents could replace pure commercial enzymes in the TaqMan qPCR assay, one of the most commonly used gold standards for molecular diagnostic procedures. A previously described molecular diagnostic assay for Zika virus detection [27] was used as an exemplar, albeit with synthetic DNA, instead of RNA, templates. Defined numbers of Taq DNA polymerase cellular reagents or standard amounts (2.5 units according to the manufacturer) of commercial Taq DNA polymerase were used to amplify the same number of template copies. Polymerase activities were compared by measuring the respective Cq values for the same number of templates. Some 2 x 10^7^ bacteria bearing Taq DNA polymerase demonstrated similar amplification efficiencies, detection limits, C_q_ values (time to detection), signal amplitudes, and absence of non-specific signals as added, 2.5 units of purified commercial Taq DNA polymerase (**Fig 1**). Most importantly, nucleic acid degradation was not evident in assays carried out with cellular reagents, likely due to inhibition and/or degradation of endogenous nucleases upon thermal cycling. Just as the preparation of thermostable enzymes in mesophilic cells is often abetted by the denaturation or aggregation of non-thermostable proteins [32], the same phenomenon can assist with molecular diagnostics.

**Fig 2:**
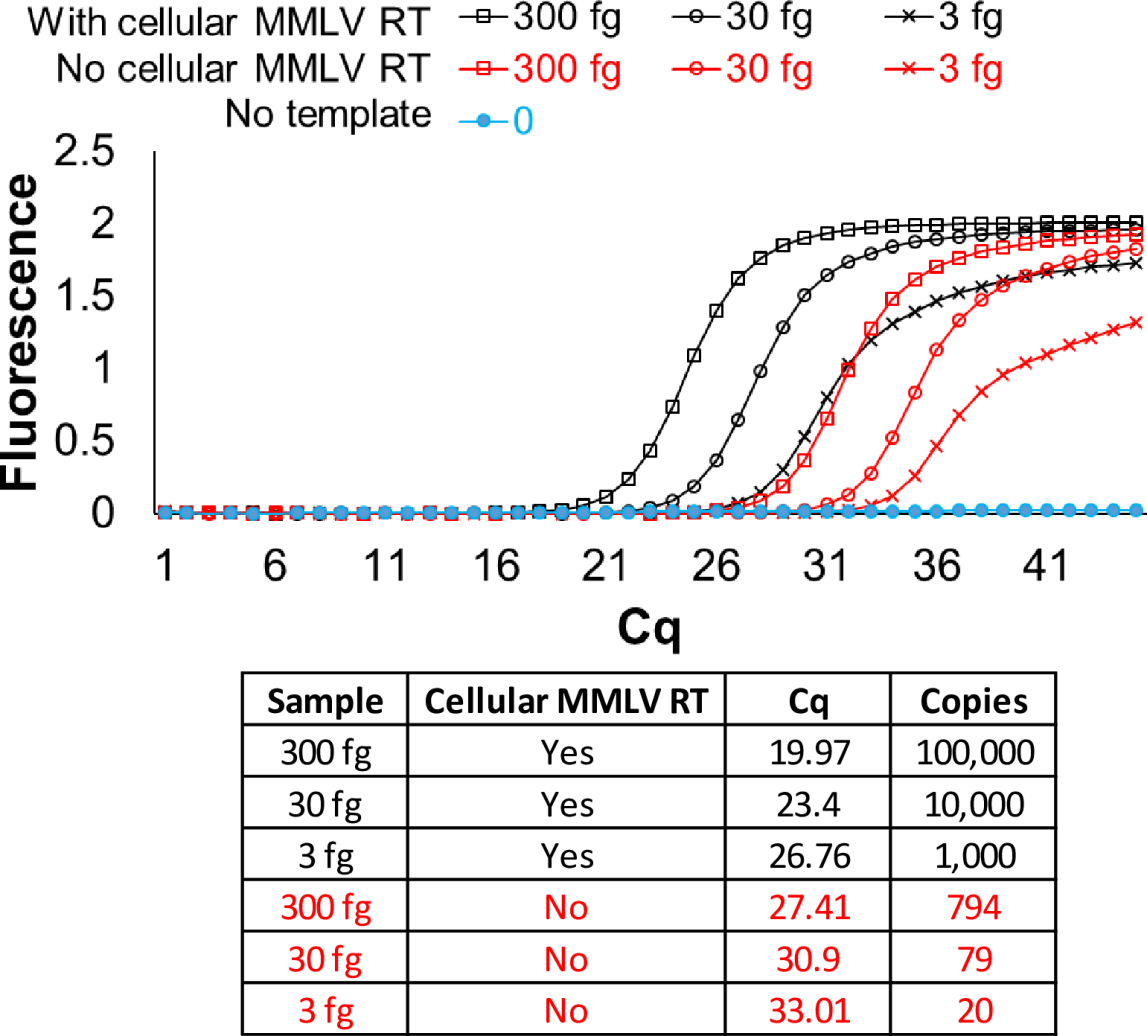
RNA detection by two-step reverse transcription TaqMan qPCR using cellular reagents for MMLV RT and Taq DNA polymerase. Indicated copies of synthetic RNA template derived from Zika virus genomic sequence were tested using 2 x 10^7^ cells each of MMLV RT and Taq DNA polymerase lyophilized cellular reagents. Amplification was assessed in real-time by measuring increase in TaqMan probe fluorescence over time. Representative amplification curves generated using the “Abs quant” analysis in the LightCycler 96 software are presented. Color of the traces indicate presence (black traces) or absence (red traces) of MMLV RT cellular reagents, or the absence of templates (blue traces). The corresponding derivation of template copies from Cq analyses are tabulated. Cq values were converted to template copies using standard curve analyses of the same RNA samples with commercial qRT-PCR master mix (**S8 Fig**).

Single enzyme cellular reagents clearly were able to be used as molecular diagnostics, raising the possibility that multiple enzymes that worked in parallel could be delivered via cells. We attempted to set up a TaqMan qPCR assay starting with RNA templates and cellular reagents. RNA templates were first reverse transcribed into complementary DNA (cDNA) using MMLV reverse transcriptase-expressing lyophilized bacteria, and then without further purification TaqMan qPCR was carried out using Taq DNA polymerase cellular reagents. The two-step cellular reagent protocol could unambiguously detect templates bearing portions of the Zika virus with C_q_ values that correlated closely with the expected 10-fold differences between various samples (**Fig 2**). RNA samples that were not subjected to reverse transcription also yielded detectable C_q_ values but these were 6.5 to 7.5 units higher than the C_q_ values obtained with the cDNA samples, as was expected in the absence of any explicit DNase treatment. Non-specific signal in the absence of templates was not observed.

**Fig 3:**
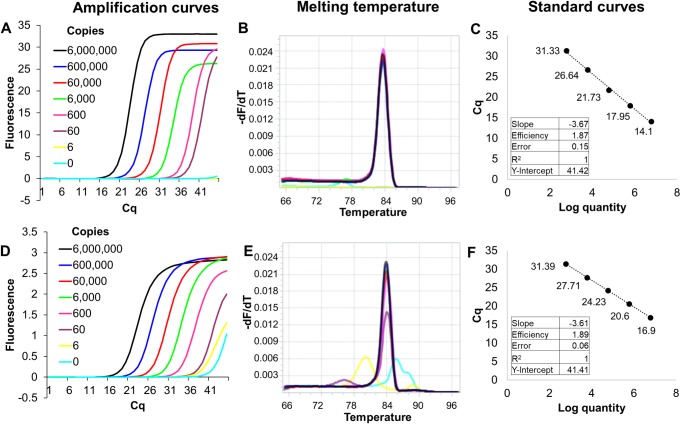
EvaGreen qPCR analysis using KlenTaq DNA polymerase expressing cellular reagents. Indicated copies of synthetic *Chlamydia trachomatis* DNA template were amplified by PCR using 0.2 μL of pure commercial KlenTaq DNA polymerase (panels a, b, and c) or 2 x 10^7^ cells of KlenTaq cellular reagents (panels d, e, and f). Amplicon accumulation was assessed in real time by measuring increase in EvaGreen fluorescence. Panels a and d depict representative amplification curves generated using the “Abs quant” analysis in the LightCycler 96 software. Colors of the curve traces indicate starting numbers of template copies–black: 6x10^6^ template copies; blue: 6x10^5^ template copies; red; 6x10^4^ template copies; green: 6x10^3^ template copies; pink: 600 template copies; purple: 60 template copies; yellow: 6 template copies; and cyan: no templates. Taken together, these curves demonstrate the real-time kinetics of PCR amplification. Since EvaGreen is a non-specific DNA intercalating dye, the fidelity of amplicon generation was verified by determining their melting temperatures (panels b and e) using the “Tm calling” analysis protocol in the LightCycler 96 software. Color coding of the curves is the same as in panels a and d. The overlapping melting temperature peaks of amplicons generated from 6 x 10^6^ to 60 copies of templates are indicative of correctly amplified PCR products. Amplification curves observed in the presence of 0 to 6 template copies are non-specific as evident from their different melting temperatures peaks of these amplicons. Standard curve analyses performed using the “Abs quant” protocol in the LightCycler 96 software are depicted in panels c and f, respectively, and data for amplification efficiency, linearity, and error are tabulated as insets.

**Fig 4:**
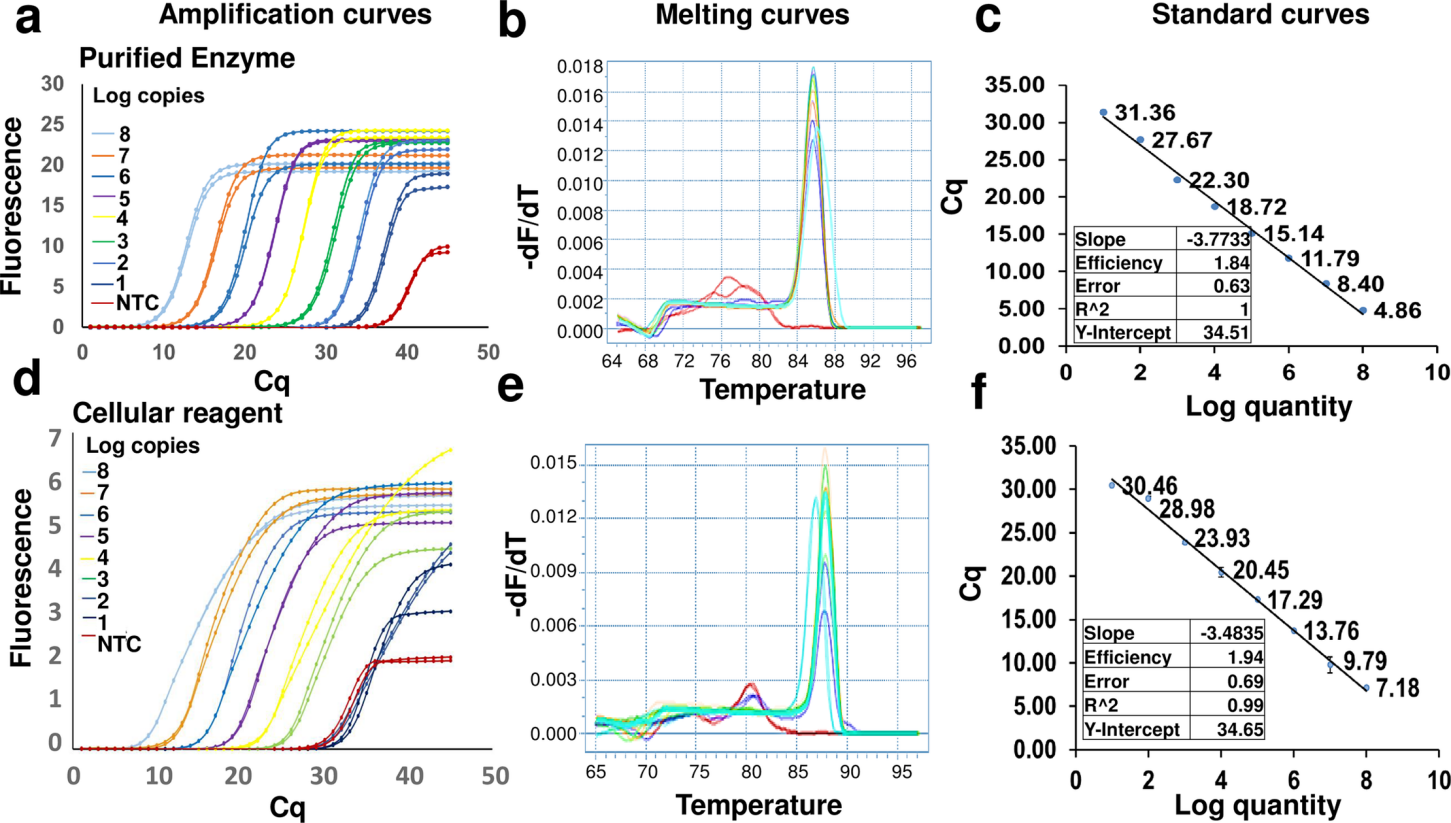
EvaGreen qPCR analysis using RTX Exo- DNA polymerase expressing cellular reagents. Indicated copies of synthetic Zika virus derived DNA template were amplified by PCR using 80 ng of pure RTX Exo- DNA polymerase (panels a, b, and c) or 2 x 10^6^ cells of RTX Exo- cellular reagents (panels d, e, and f). Amplicon accumulation was assessed in real time by measuring increase in EvaGreen fluorescence. Representative amplification curves using 10^8^, 10^7^, 10^6^, 10^5^, 10^4^, 10^3^, 10^2^, 10 and 0 template DNA copies are shown in panels a and d. ‘NTC’ refers to no template control. These curves were generated using the “Abs quant” analysis protocol in the LightCycler 96 software. The corresponding amplicon melting temperature analyses performed using the “Tm calling” protocol in the LightCycler 96 software are shown in panels b and e. The melting temperature peaks of target-derived amplicons are distinct from those of non-specific amplicons generated in the absence of templates. Standard curve analyses performed using the “Abs quant” protocol in the LightCycler 96 software are depicted in panels c and f. Standard curve analyses data for comparing amplification efficiency, linearity, and error are tabulated as insets.

**Fig 5:**
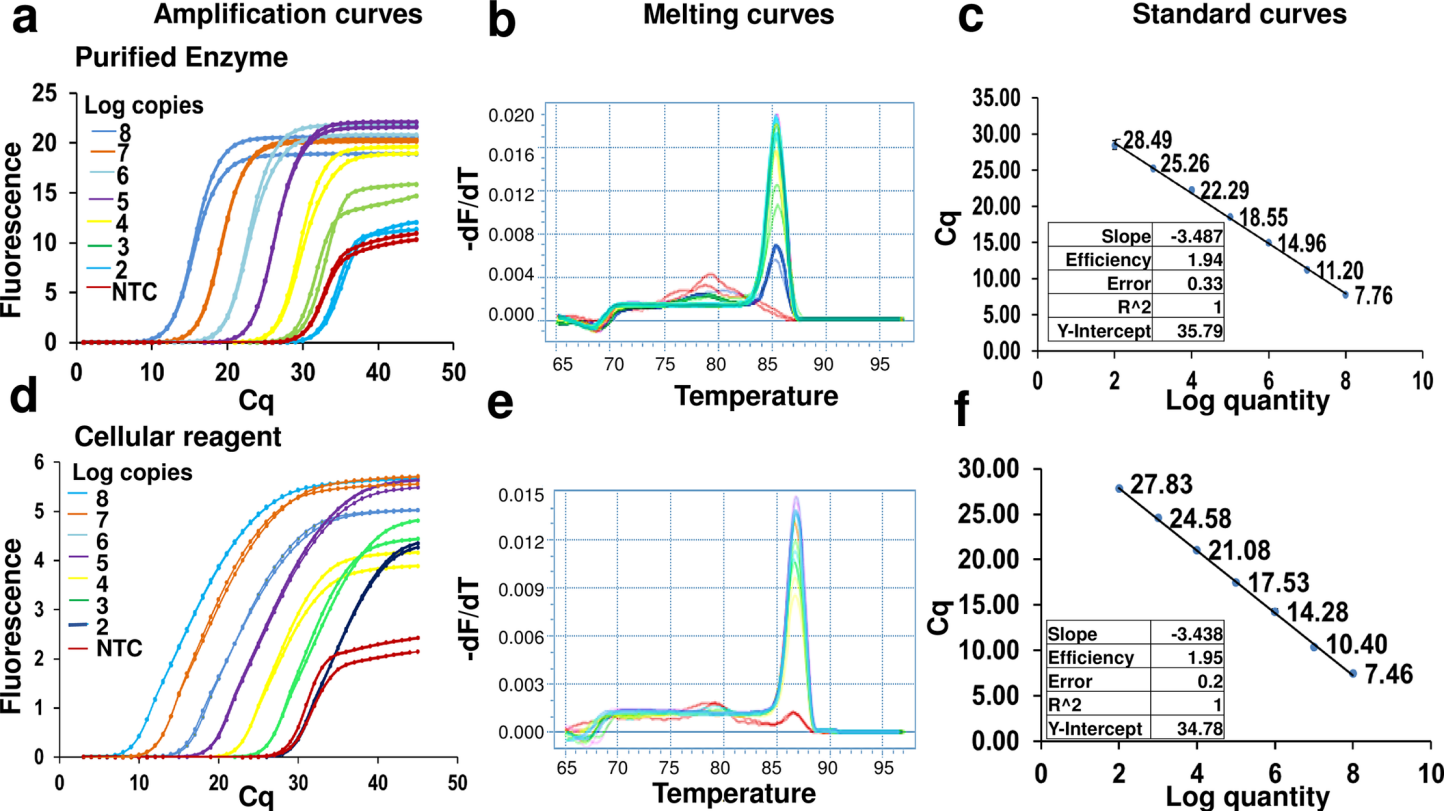
EvaGreen qRT-PCR analysis using RTX Exo- DNA polymerase expressing cellular reagents. Indicated copies of synthetic Zika virus derived RNA template were amplified by RT-PCR using 80 ng of pure RTX Exo- DNA polymerase (panels a, b, and c) or 2 x 10^6^ cells of RTX Exo- cellular reagents (panels d, e, and f). Amplicon accumulation was assessed in real time by measuring increase in EvaGreen fluorescence. Representative amplification curves using 10^8^, 10^7^, 10^6^, 10^5^, 10^4^, 10^3^, 10^2^, 10 and 0 template RNA copies are shown in panels a and d. These curves were generated using the “Abs quant” analysis protocol in the LightCycler 96 software. ‘NTC’ refers to no template control. The corresponding amplicon melting temperature analyses performed using the “Tm calling” protocol in the LightCycler 96 software are shown in panels b and e. The melting temperature of non-specific amplicons generated in the absence of templates is distinct from target-derived amplicons. Standard curve analyses performed using the “Abs quant” protocol in the LightCycler 96 software are depicted in panels c and f. Standard curve analyses data for comparing amplification efficiency, linearity, and error are tabulated as insets.

Finally, since many diagnostic protocols use DNA intercalating fluorophores for measuring amplicon accumulation we developed EvaGreen qPCR mixes containing lyophilized BL21 cellular reagents expressing KlenTaq or RTX Exo- DNA polymerase. In standard curve analyses of *Chlamydia trachomatis* DNA templates, 2 x 10^7^ cells of KlenTaq polymerase-expressing cellular reagents demonstrated similar amplification efficiencies, Cq values, and detection limits as the standard pure enzyme amount of 0.2 μL / reaction suggested by the manufacturer. Similarly, 2 x 10^6^ RTX Exo- cellular reagents demonstrated similar amplification efficiencies, C_q_ values, and detection limits with Zika virus-derived DNA templates, as optimal amount of 80 ng/ reaction of the pure enzyme (**Figs 3 and 4**). In fact, a one-step qRT-PCR mix built using cellular reagents expressing the RTX thermostable reverse transcriptase could accurately quantify Zika virus-derived RNA templates in a one-pot reaction (**Fig 5**). Despite the fact that a TaqMan probe was not involved, negligible non-specific signal was observed with either of these approaches. Moreover, target-derived amplicons could be readily identified by their characteristic melting temperatures. The slight increase in amplicon melting temperature observed in reactions containing cellular reagents might be due to an accompanying increase in ionic concentration and molecular crowding [33, 34].

Overall, these results indicate that cellular reagents are suitable for a variety of PCR formats. Under the reported conditions substitution of commercial reagents with cellular reagents in qPCR resulted in a statistically significant but minute increase of Cq by 1.6 units (**S1 Statistical Supplement**). This small difference should not be a deterrent to the adoption of cellular reagents, especially since the same number of template copies could be detected with cellular or purified commercial reagents, with no interference from non-specific amplicons.

### Isothermal nucleic acid amplification using cellular reagents

**Fig 6:**
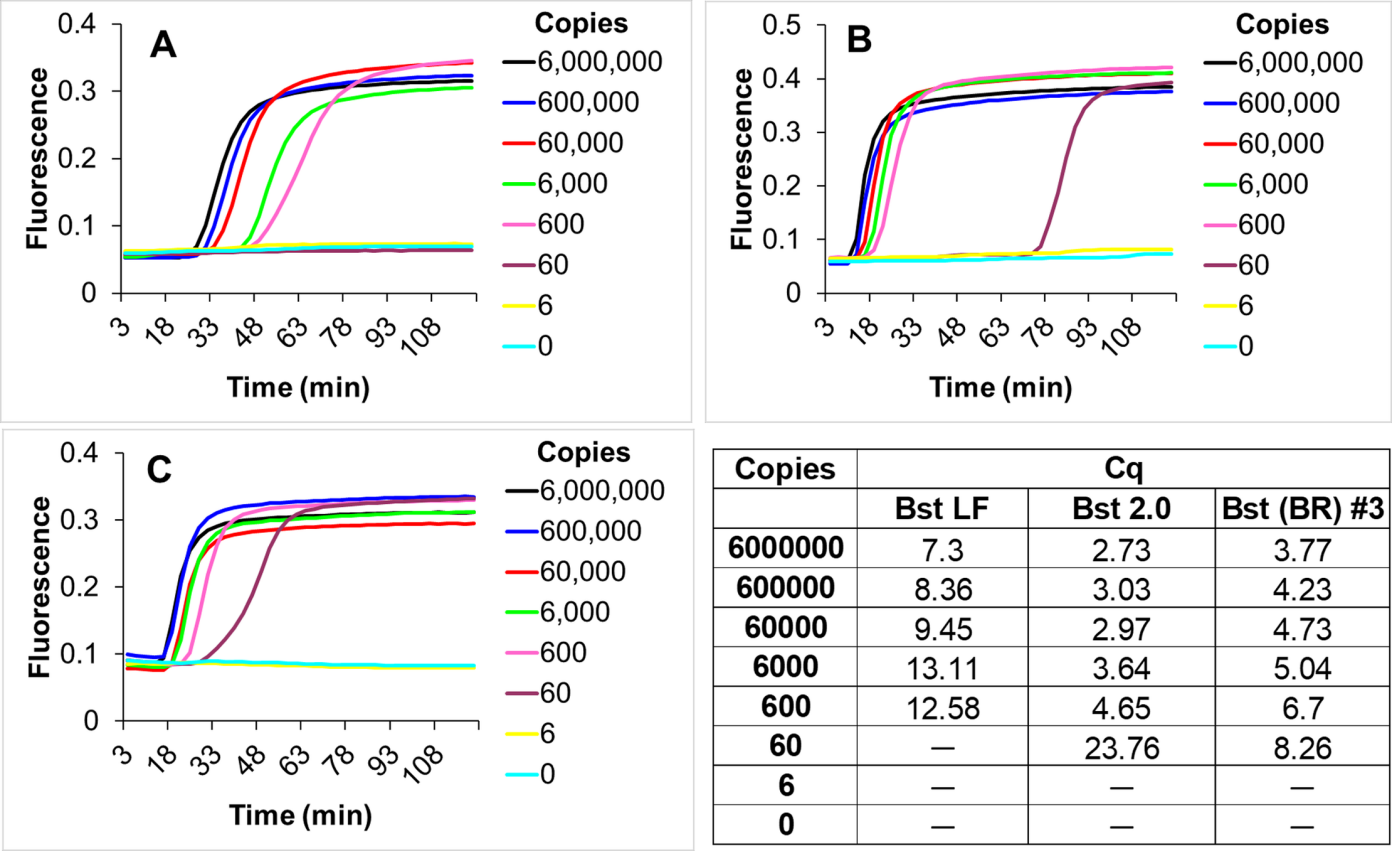
Isothermal nucleic acid amplification using Bst DNA polymerase cellular reagents. Indicated copies of synthetic DNA templates derived from human glyceraldehyde 3-phosphate dehydrogenase gene were amplified in LAMP-OSD reactions using 16 units of pure Bst-LF (panel a), 16 units of pure Bst 2.0 (panel b), or 2 x 10^7^ cells of Bst-LF cellular reagents (panel c). Amplicon accumulation was assessed in real time by measuring increase in OSD fluorescence. Representative raw fluorescence amplification curves are depicted in black (6 x 10^6^ template copies), blue (6 x 10^5^ template copies), red (6 x 10^4^ template copies), green (6 x 10^3^ template copies), pink (600 template copies), purple (60 template copies), yellow (6 template copies), and cyan (0 templates). Cq values obtained using pure commercial Bst-LF (panel a), pure commercial Bst 2.0 (panel b), and Bst-LF cellular reagent (CR) (panel c) are tabulated. Unlike PCR, LAMP is a complex continuous amplification process in which Cq does not always correlate linearly with template copies.

Beyond PCR, it should be possible to carry out other reactions using cellular reagents. To demonstrate the general utility of the concept, cellular reagents expressing Bst-LF, the *Bacillus stearothermophilus* strand displacing DNA polymerase (large fragment) commonly used for isothermal nucleic acid amplification reactions were assayed for their ability to carry out LAMP-OSD (**S9 Fig**). Similar to 16 units of pure commercial Bst 2.0 enzyme, 2 x 10^7^ Bst-LF cellular reagents could amplify as few as 60 copies of human glyceraldehyde-3-phosphate dehydrogenase gene target, within 60 min (**Fig 6**). Although, Bst 2.0 is an *in silico* designed polymerase engineered for greater amplification speed and yield than Bst-LF (NEB), 2 x 10^7^ cellular Bst-LF reagents were only ~2 Cq slower than 16 units of commercial Bst 2.0 (**S1 Statistical Supplement**). This level of performance by Bst-LF cellular reagents is especially impressive considering the fact that most users typically apply only 8 units of Bst 2.0 per reaction to reduce reaction cost [35]. However, use of 10-fold lower amounts of Bst-LF cellular reagents failed to generate any LAMP-OSD signals (data not shown). Nonetheless, as long as a sufficient number of bacteria were added, the production process for the creation of lyophilized cellular reagents was robust to perturbations such as bacterial sub-culture initiation density, optical density at logarithm phase induction of protein expression, alteration of expression platform, and induction duration (**S10 Fig**). Furthermore, similar to Taq DNA polymerase, Bst DNA polymerase cellular reagents were stable for at least 3 weeks when stored at temperatures as high as 42°C (**S11 Fig**).

### Cellular reagents for molecular and synthetic biology

Given that cellular reagents were robust in various amplification reactions and formats, we were curious to determine to what extent they could be used in other contexts. Molecular and synthetic biology techniques are currently heavily reliant on the activity of pure enzymes. To demonstrate the possibilities for facile, multi-enzyme cloning procedures with cellular reagents, we created a set of cells that would contain either enzymes or templates as input for one of the most commonly used methods for cloning, Gibson assembly of DNA fragments [24]. Gibson assembly can be used to create vectors from two or more DNA fragments in a one-pot reaction by relying on complementary overlaps created by T5 exonuclease degradation. Once complementary strands have come together, DNA polymerase is used to fill gaps, and Taq DNA ligase seals the remaining nicks to create the new vector.

To most simply carry out Gibson assembly with cellular reagents we merely lyophilized three cell lines that expressed Taq DNA polymerase, Taq DNA ligase, and T5 exonuclease, respectively. We first attempted the simple assembly of a double-stranded gBlock insert (representing a portion of the *Aedes albopictus* ribosomal protein S7 gene or the *E*. *coli yai*O gene) with 30 bp overlaps corresponding to each end of gel-purified linearized vector, pCR2.1 TOPO (Thermo Scientific) using only cellular reagents. Vector and insert were mixed in equimolar ratio in 1X Gibson assembly buffer and then treated with purified or cellular reagents for Gibson cloning. We hypothesized that endogenous nucleases in BL21 cellular reagents would linearize DNA fragments and prevent recovery of engineered DNA, and therefore opted to use cellular reagents prepared in a Top10 *E*. *coli* strain that lacked endonuclease I and recA1. However, initial experiments with these Top10 cellular reagents failed to yield successful DNA assemblies. Optimization experiments revealed that a 10 min pre-heating of Taq DNA polymerase and Taq DNA ligase cellular reagents at 75°C significantly improved Gibson assembly (**S2 Table**). When DNA parts were mixed with treated cellular reagents comprising 2 x 10^7^ Taq DNA ligase cells, 10^6^ Taq DNA polymerase cells, and 10^5^ T5 exonuclease cells (**S2 Table**) the Gibson cloning procedure was successful, producing 48–60% of the number of colonies observed with a commercial enzyme master mix containing 0.08 units of T5 exonuclease, 0.5 units of Phusion DNA polymerase, and 80 units of Taq DNA ligase. Background assembly was similar for both cellular and commercial reagents. That said, while most clones assembled by pure enzymes were correct only 50% of the plasmids assembled by cellular reagents had the correct sequence; the remainder were re-circularized vectors.

**Fig 7:**
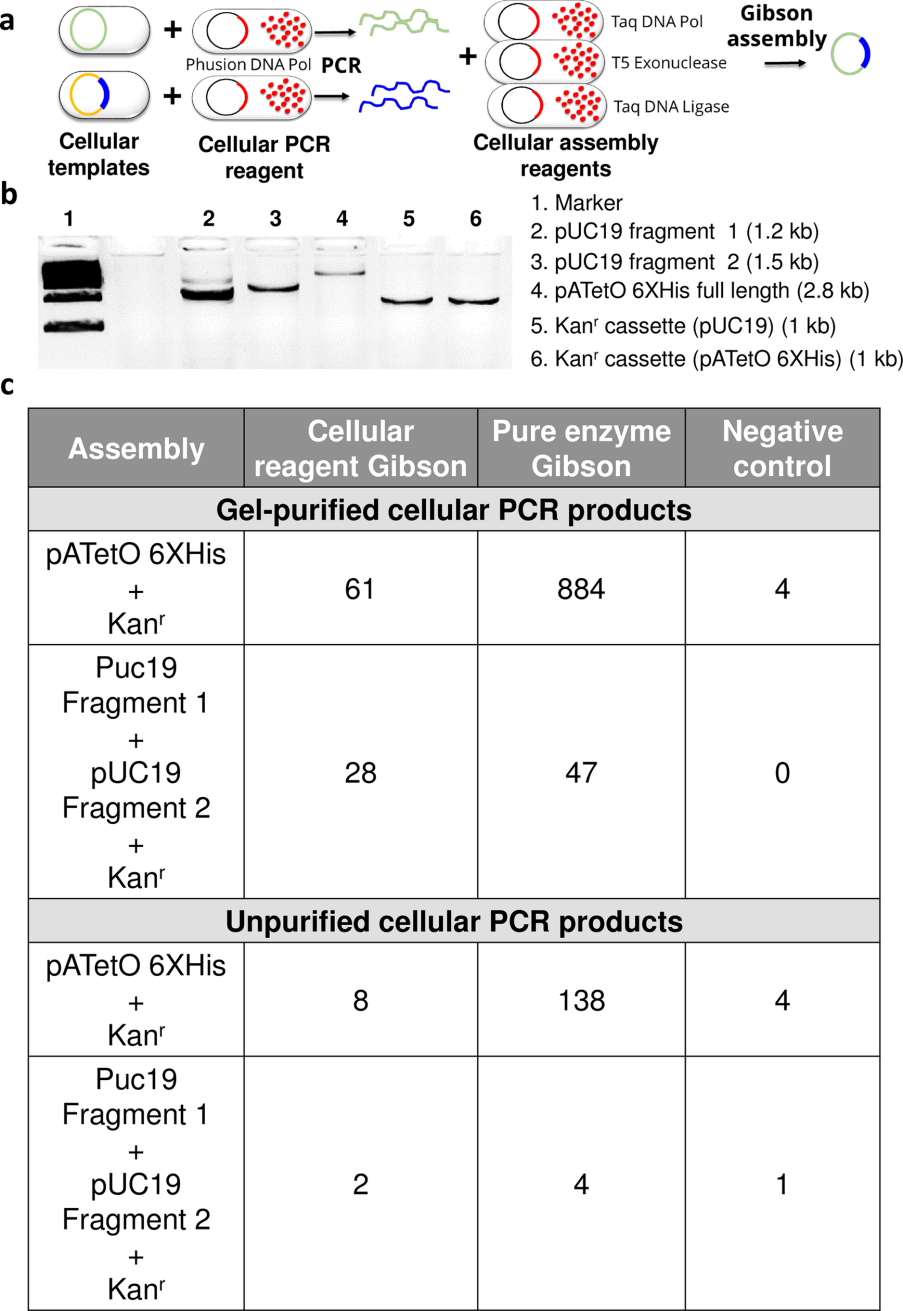
PCR and Gibson assembly using cellular reagents. (a) Schematic depicting cellular PCR followed by cellular Gibson assembly for constructing new plasmids. Bacteria harboring target plasmids are mixed with polymerase-expressing cellular reagents and PCR is initiated by adding appropriate primers, buffer, and dNTP. The resulting PCR products are incubated with cellular reagents expressing Gibson assembly enzymes–Taq DNA polymerase, Taq DNA ligase, and T5 exonuclease–to assemble the new construct. (b) Cellular PCR amplification of vector and insert fragments directly from *E*. *coli* bacteria bearing target DNA plasmids using 2 x 10^7^ cells of Phusion cellular reagents. Assembly parts include: (i) “pATetO 6XHis full length” vector for two part assembly with Kan^r^ cassette bearing appropriate overlapping ends, and (ii) “pUC19 Fragments 1 and 2” for three part assembly with Kan^r^ cassette whose ends overlap with pUC19 vector fragments. (c) Gibson assembly of agarose gel purified and unpurified cellular PCR products using pure or cellular Gibson assembly reagents. In “negative control” samples the PCR products were incubated in Gibson reaction buffer without pure or cellular Gibson enzymes. “pATetO 6XHis + Kan^r^”represents a two part Gibson assembly while “Puc19 Fragment 1 + pUC19 Fragment 2 + Kan^r^” represents a three-part Gibson assembly. Representative number of clones recovered in each case in the presence of both ampicillin and kanamycin are reported.

Despite an increased propensity for incorrect assembly, cellular Gibson assembly is nonetheless a highly enabling technology that stands to significantly reduce costs for and increase accessibility to a core tool for synthetic biology. To further bolster the appeal and utility of cellular Gibson assembly we sought to combine amplification and assembly with cellular reagents. As a first step, cellular Phusion reagents were used to amplify linear vector and insert fragments directly from two different bacterial strains (**Fig 7**). The kanamycin resistance cassette from the donor plasmid pJLsfGFP and the entire vector backbone of ampicillin-resistant pATetO 6XHis plasmid were amplified with the inclusion of 30 bp overlaps. Following agarose gel purification of the DNAs produced by amplification with cellular reagents, Gibson assembly with either cellular or purified reagents yielded 61 and 884 clones, respectively, that were jointly resistant to both ampicillin and kanamycin; all were verified by sequencing to be correctly assembled (**Fig 7**). We similarly attempted a three-part assembly with DNAs amplified by cellular reagents by dividing pUC19 into two linear fragments with 30 bp overlaps prior to purification and assembly. The three part assembly yielded 28 clones, all of which were correctly assembled (**Fig 7**). The improvement in the fraction of correctly assembled vectors in this example (100%) relative to the previous example with gBlock insertion was likely due to the fact that correctly assembled vectors could be directly selected via antibiotic resistance.

Finally, we attempted to carry out the same assemblies without DNA purification, using only cellular Gibson reagents. We obtained eight and two colonies, respectively, all of which proved to be the correct assembly (**Fig 7**).

## Discussion

Currently, widespread application of molecular biology techniques in research, development of diagnostics, and education is costly and often unaffordable for many laboratories and schools especially in resource poor countries. In addition to investments in equipment, the costs of consumable protein and enzyme reagents is a major roadblock to wider dissemination and use of molecular techniques [15, 16]. By demonstrating the feasibility of using cellular reagents in lieu of pure enzymes for routine applications, such as nucleic acid synthesis, Gibson assembly of DNA, and nucleic acid diagnostics, such as TaqMan qPCR and LAMP, we have created a relatively inexpensive reagent toolbox. Seamless substitution of ready-to-use cellular reagents that do not require any additional lysis or extraction steps in fungible molecular biology reactions and assays could potentially radically alter paradigms for sample and assay preparation. The resulting cost reduction could open the way to synthetic and molecular biology innovations that would more readily transition directly to research and to resource poor settings. With further optimizations, standardization of manufacturing, and larger-scale validations, it is also conceivable that cellular reagents might find use in clinical diagnostics. Indeed other complex reagents, such as infectious bacteriophages, have been approved by the FDA for clinical use in identification of bacteria and their antibiotic resistance status [36].

Compared to current technologies for the production and distribution of purified protein reagents, cellular reagents present several advantages. These include: (i) lower production time and cost due to the elimination of protein purification; (ii) simplified quality control during production in which optimized culture density (measured as A_600_) is a convenient metric for ensuring uniformity of performance; (iii) favorable yield for many small to medium scale applications (1 ml culture = 150 qPCR or isothermal amplification reactions); (iv) cheaper storage and transport without a cold chain; (v) seamless integration of cellular reagents into many different molecular biology technologies due to easy access to the enzyme payload without additional bacterial lysis steps; and (vi) negligible declines in assay performance or outcomes when using cellular reagents. Overall, these advantages make cellular reagents significantly cheaper to produce, store, distribute, and use. Furthermore, since the cellular reagent production process involves considerably fewer procedures and equipment it should be easier to adopt by scientists, clinicians, or companies in a small region. It is also conceivable that the aforementioned advantages in cost and process frugality offered by cellular reagents could provide benefits for niche applications such as research at remote terrestrial or extraterrestrial stations [37] where transport of elaborate purification equipment or liquid reagents can be difficult and expensive.

There has recently been a surge of interest in ‘in vitro biology’ [38–40], with *in vitro* transcription and translation (IVTT) reactions being used for diagnostic applications [41] and for the production of many new proteins [42–45]. Simplification of the traditionally labor and cost intensive procedures for preparation and storage of *E*. *coli* IVTT extracts is key to wider and newer applications [46]. For instance, lyophilization of lysed *E*. *coli* extracts has allowed high-density storage and improved shelf-life [47]. Our work now begins to demonstrate that perhaps IVTT procedures might be further simplified by eliminating lysis and basal purification. The interest in *in vitro* biology overlaps significantly with the continuing adoption of synthetic biology methods for both research and education [48, 49], and the demonstration that cellular reagents can be used for Gibson assembly points the way to a new paradigm for making constructs in which a variety of ‘cellular templates’ might be distributed along with cellular reagents to create entirely new constructs. Into the future, we believe the cellular reagent platform can be expanded to offer additional enzymatic activities as well as alternate cellular environments such as those offered by eukaryotes like *Pichia pastoris* and *Saccharomyces cerevisiae* [50].

## Supporting information

S1 Fig Endpoint PCR analysis using fresh culture of RTX Exo- polymerase expressing cellular reagents. Control PCR amplifications performed using pure RTX Exo- polymerase and KOD polymerase are shown in the top panel. Bottom panel depicts PCR products generated using fresh (non-lyophilized) cellular reagents.
(PDF)

S2 Fig PCR amplification efficiencies of lyophilized or frozen RTX Exo- polymerase expressing cellular reagents and purified RTX Exo- polymerase. Synthetic DNA templates derived from *Chlamydia trachomatis* 16S rRNA gene were amplified using purified or cellular RTX Exo- reagents. Amplicon accumulation was measured in real time using EvaGreen fluorescent dye intercalation. Amplification curves generated by RTX Exo- DNA polymerase are shown in ***A*** in deep purple (with 10^8^ copies of template) and in light purple (without template). Amplification curves generated by BL21 DE3 bacteria that are not expressing any exogenous polymerases are shown in dark green (with 10^8^ copies of template) and in light green (without template). Amplicon melting temperature peaks generated by performing “Tm calling” analysis using the LightCycler 96 software are depicted on the right. Color coding is the same as in the amplification curves. Target-derived amplicons can be readily distinguished from non-specific products by their distinct melting peaks. The high amplitude of the dark green curve in the top left panel in ***A*** is an artifact of data analysis. These amplification curves generated by the “Abs quant” protocol in the LightCycler 96 software depict the rate of change of the rate of change of fluorescence. BL21 DE3 cells that do not express RTX polymerase only yield background fluorescence with or without template as evident from the raw fluorescence curves depicted in ***B***. The difference in the background level of fluorescence of frozen versus lyophilized cells might be a reflection of the lyophilization-induced alterations in bacterial cells.
(PDF)

S3 Fig qPCR analysis using lyophilized RTX Exo- expressing cellular reagents stored at room temperature for ~80 days. Real-time qPCR amplification curves for 10^6^ (red), 10^7^ (cyan), 10^8^ (green), and 0 (blue) copies of synthetic *Chlamydia trachomatis* 16S rDNA templates are depicted in panel a. Amplicon accumulation was measured as increase in fluorescence of the intercalating dye EvaGreen. Melting curve analysis of amplicons was performed using the “Tm calling” protocol in the LightCycler 96 software (panel b). This analysis allows identification and distinction of target-derived amplicons whose Tm peak is distinct from the melting temperature of non-specific amplicons. Color coding of the melting peaks is the same as that of the amplification curves. Cq of detecting different template copies is plotted as a bar graph in panel c. Standard curve analysis performed using the “Abs quant” protocol in the LightCycler 96 software is depicted in panel d.
(PDF)

S4 Fig Assessment of bacterial viability in cellular reagents. BL21 *E*. *coli* expressing Taq DNA polymerase were lyophilized in either 1X PBS or in 1X PBS supplemented with 0.1M trehalose. After 3 days of storage at ambient temperature, the lyophilized cellular reagents were rehydrated in 30 μL water and half of the material was spread plated on Luria Bertani agar plates. Images of these plates were taken after overnight incubation at 37°C. Only bacteria that were lyophilized in the presence of trehalose retained viability. Cellular reagents lyophilized without trehalose do not remain viable.
(PDF)

S5 Fig Overlap extension assays to evaluate enzyme accessibility in cellular reagents. BL21 *E*. *coli* cells overexpressing Taq DNA polymerase were washed in PBS and assessed for enzyme activity in three different conditions: fresh cells (FR), cells frozen at -80°C (FO), or lyophilized (L) cells. Cells (C) were tested isothermally by single step overlap extension assays at four different temperatures– 37°C, 42°C, 65°C, and 75°C. The PBS supernatants (S) leftover after pelleting fresh (S^FR^) or frozen (S^FO^) cells were also tested for polymerase activity. Overlap extension performed using pure (P) commercial Taq DNA polymerase served as the positive control. Reactions performed in the presence of oligonucleotide templates are labeled ‘Templates’. Negative controls lacking templates are denoted as ‘NTC’. All overlap extension products (indicated by ‘*’) were analyzed by agarose gel electrophoresis. Overlap extension template oligonucleotides (O; indicated with ‘#’) were analyzed as controls.
(PDF)

S6 Fig Microscopic examination of cellular reagents. Freshly cultured *E*. *coli* cells overexpressing RTX DNA polymerase were washed and resuspended either in 1X PBS (a) or in water (b) prior to Gram staining and microscopic imaging under oil immersion and a 100X objective lens. Aliquots of these cells were also lyophilized and then rehydrated with water prior to microscopy. Cells lyophilized in 1X PBS are depicted in panel c while lyophilized cells examined after heat treatment are depicted in panels d (cells lyophilized in 1X PBS) and e (cells lyophilized in water).
(PDF)

S7 Fig Storage stability of Taq DNA polymerase cellular reagents at elevated temperatures. Taq DNA polymerase expressing cellular reagents stored with desiccant at 25°C, 37°C, or 42°C were tested for activity by using 2 x 10^7^ cells per reaction in endpoint PCR. Products were analyzed by gel electrophoresis and compared to PCR performed using 2.5 units of pure commercial Taq DNA polymerase. Activity of cellular reagents after 21 days of storage are depicted.
(PDF)

S8 Fig Standard curve analysis of Zika virus RNA using commercial one-pot qRT-PCR master mix. **A.** Zika virus derived synthetic RNA template was analyzed by one-pot qRT-PCR using the Evoscript RNA Probes Master mix (Roche) according to the manufacturer’s instructions. Briefly, indicated RNA template copies were added to 1 X qRT-PCR master mix supplemented with 800 nM each of Zika 4481_F and Zika 4552c forward and reverse primers, and 200 nM of Zika 4507c-FAM TaqMan probe. PCR reactions were first incubated at 60°C for 30 min to allow reverse transcription. The reactions were then incubated at 95°C for 10 min prior to executing 45 cycles of 15 sec at 95°C and 30 sec at 55°C. Amplicon accumulation was measured as increase in TaqMan probe fluorescence. Amplification curves obtained using indicated copies of template RNA are depicted. These curves were generated using “Abs quant” analysis protocol in the LightCycler 96 software. **B.** Standard curve analysis of real-time amplification data shown in panel A.
(PDF)

S9 Fig LAMP-OSD schematic. LAMP uses 2 inner (FIP and BIP) and 2 outer (F3 and B3) primers specific to 6 blocks of target sequences designated as B3, B2, B1, F1c, F2c and F3c. F2 sequence in FIP (F1c-F2) initiates amplification by Bst DNA polymerase (Stage I). F1c sequence in FIP self-primes subsequent amplification. Similarly, BIP (B1c-B2) initiates DNA synthesis by binding to B2c. F3 and B3 primer-initiated DNA synthesis displaces preceding inner primer-initiated strands, which serve as templates for primer-initiated strand displacement DNA synthesis (Stage II). 3′-ends of the resulting single-stranded, dumbbell-shaped amplicons (Stage III) are extended by Bst polymerase to form hairpins (Stage IV). Inner primers hybridize to the single-stranded loops and initiate another round of strand displacement synthesis that opens the original hairpin to form a concatemerized amplicon containing a self-priming 3′-end hairpin (Stage V). The ensuing continuous amplification (initiated both by new inner primers and by self-priming) generates increasingly complex, double-stranded concatameric amplicons containing self-priming hairpins and single-stranded loops to which the OSD probe hybridizes. “c”: denotes complementary target sequences. F and Q on the OSD denote fluorophore and quencher, respectively. OSD probe is denoted in terms of numbered domains, each of which represents a short fragment (usually <12 nt) of DNA sequence in an otherwise continuous oligonucleotide strand. Single stranded toeholds are numbered in red. Complementarity between numbered OSD domains is denoted by a single prime symbol.
(PDF)

S10 Fig Effect of culture conditions on performance of cellular reagents. Amplification efficiencies of lyophilized cellular reagents expressing Bst-LF DNA polymerase were tested in LAMP-OSD assays using indicated template copies. LAMP amplicon accumulation was measured in real-time using fluorogenic OSD probes. Cq values (time-to-signal) at different template copies determined using the “Abs quant” analysis protocol in the LightCycler 96 software are depicted.
(PDF)

S11 Fig Storage stability of Bst-LF cellular reagents at elevated temperatures. Amplification efficiencies of lyophilized cellular reagents expressing Bst-LF DNA polymerase were tested in LAMP-OSD assays using indicated template copies. LAMP amplicon accumulation was measured in real-time using fluorogenic OSD probes. Amplification curves obtained with either 60,000 (full traces labeled “(+)”) or 0 (dashed traces labeled “(-)”) copies of *gapd* templates are depicted. Amplification curves were generated using the “Abs quant” analysis protocol in the LightCycler 96 software. Bst-LF cellular reagents were either tested immediately after lyophilization (black traces labeled “Same day”) or after storage with desiccants for 21 days at 25°C (green traces labeled “25 C”), 37°C (pink traces labeled “37 C”), or 42°C (blue traces labeled “42 C”).
(PDF)

S1 Table Oligonucleotide and template sequences used in the study. (PDF)

S2 Table Cellular reagent Gibson assembly with or without heat treatment of cellular reagents. (PDF)

S1 Statistical Supplement Cellular Reagents for Diagnostics and Synthetic Biology Statistical Supplement. (PDF)
