# Supplementary material for: Cellular reagents for diagnostics and synthetic biology
Source: PLoS One. 2018 Aug 15;13(8):e0201681. doi: 10.1371/journal.pone.0201681 (PMC6093680; doi:10.1371/journal.pone.0201681)
Supplement: S1 Fig — Control PCR amplifications performed using pure RTX Exo- polymerase and KOD polymerase are shown in the top panel. Bottom panel depicts PCR products generated using fresh (non-lyophilized) cellular reagents. (PDF) [file pone.0201681.s001.pdf]

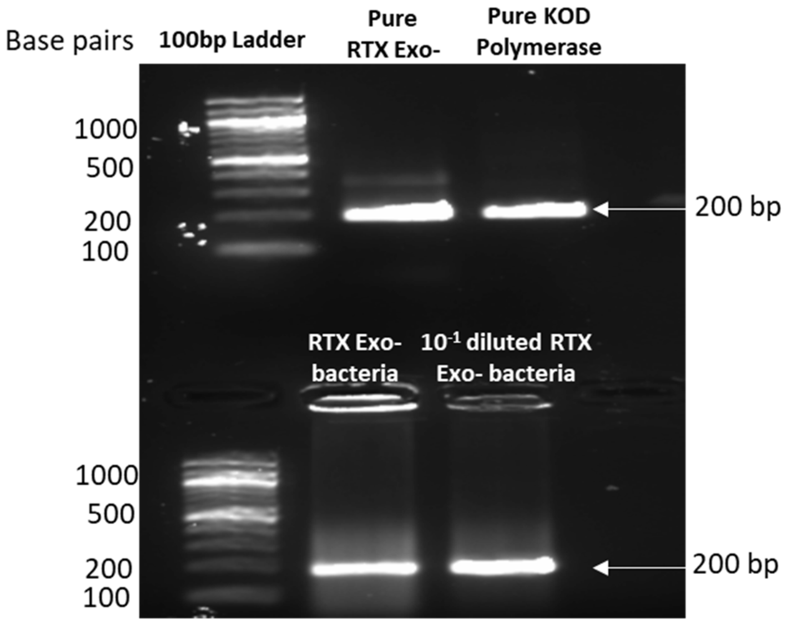

S1 Fig. Endpoint PCR analysis using fresh culture of RTX Exo- polymerase expressing cellular reagents. Control PCR amplifications performed using pure RTX Exo- polymerase and KOD polymerase are shown in the top panel. Bottom panel depicts PCR products generated using fresh (non-lyophilized) cellular reagents.
