# Supplementary material for: Cellular reagents for diagnostics and synthetic biology
Source: PLoS One. 2018 Aug 15;13(8):e0201681. doi: 10.1371/journal.pone.0201681 (PMC6093680; doi:10.1371/journal.pone.0201681)
Supplement: S2 Fig — Synthetic DNA templates derived from Chlamydia trachomatis 16S rRNA gene were amplified using purified or cellular RTX Exo- reagents. Amplicon accumulation was measured in real time using EvaGreen fluorescent dye intercalation. Amplification curves generated by RTX Exo- DNA polymerase are shown in A in deep purple (with 108 copies of template) and in light purple (without template). Amplification curves generated by BL21 DE3 bacteria that are not expressing any exogenous polymerases are shown in dark green (with 108 copies of template) and in light green (without template). Amplicon melting temperature peaks generated by performing “Tm calling” analysis using the LightCycler 96 software are depicted on the right. Color coding is the same as in the amplification curves. Target-derived amplicons can be readily distinguished from non-specific products by their distinct melting peaks. The high amplitude of the dark green curve in the top left panel in A is an artifact of data analysis. These amplification curves generated by the “Abs quant” protocol in the LightCycler 96 software depict the rate of change of the rate of change of fluorescence. BL21 DE3 cells that do not express RTX polymerase only yield background fluorescence with or without template as evident from the raw fluorescence curves depicted in B. The difference in the background level of fluorescence of frozen versus lyophilized cells might be a reflection of the lyophilization-induced alterations in bacterial cells. (PDF) [file pone.0201681.s002.pdf]

A

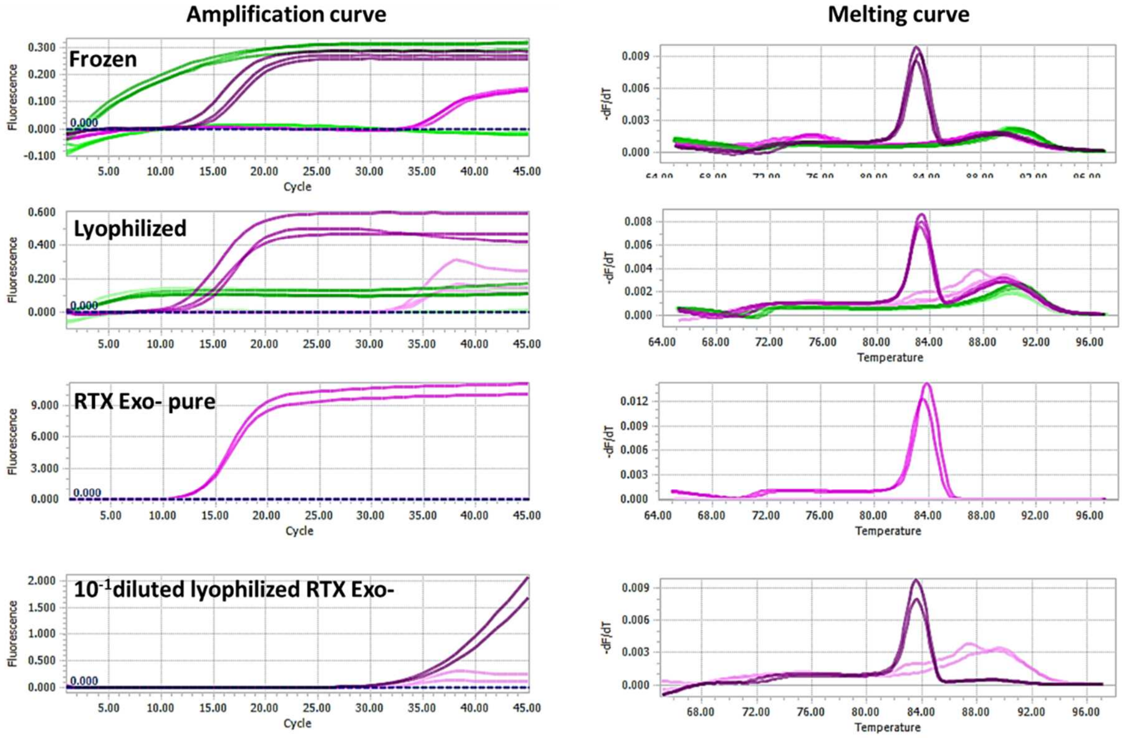

B

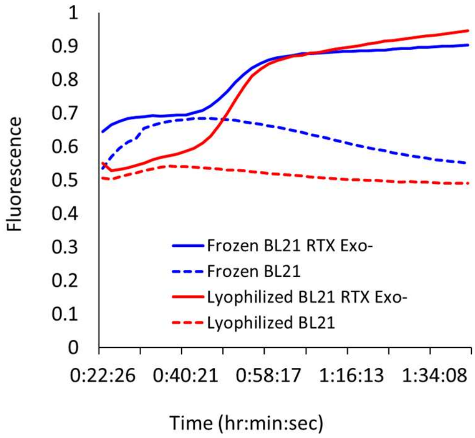

S2 Fig. **PCR amplification efficiencies of lyophilized or frozen RTX Exo- polymerase expressing cellular reagents and purified RTX Exo- polymerase.** Synthetic DNA templates derived from *Chlamydia trachomatis* 16S rRNA gene were amplified using purified or cellular RTX Exo- reagents. Amplicon accumulation was measured in real time using EvaGreen fluorescent dye intercalation. Amplification curves generated by RTX Exo- DNA polymerase are shown in ***A*** in deep purple (with 10^8 copies of template) and in light purple (without template). Amplification curves generated by BL21 DE3 bacteria that are not expressing any exogenous polymerases are shown in dark green (with 10^8 copies of template) and in light green (without template). Amplicon melting temperature peaks generated by performing "Tm calling" analysis using the LightCycler 96 software are depicted on the right. Color coding is the same as in the amplification curves. Target-derived amplicons can be readily distinguished from non-specific products by their distinct melting peaks. The high amplitude of the dark green curve in the top left panel in ***A*** is an artifact of data analysis. These amplification curves generated by the "Abs quant" protocol in the LightCyler 96 software depict the rate of change of the rate of change of fluorescence. BL21 DE3 cells that do not express RTX polymerase only yield background fluorescence with or without template as evident from the raw fluorescence curves depicted in ***B***. The difference in the background level of fluorescence of frozen versus lyophilized cells might be a reflection of the lyophilization-induced alterations in bacterial cells.
