# Supplementary material for: Cellular reagents for diagnostics and synthetic biology
Source: PLoS One. 2018 Aug 15;13(8):e0201681. doi: 10.1371/journal.pone.0201681 (PMC6093680; doi:10.1371/journal.pone.0201681)
Supplement: S3 Fig — Real-time qPCR amplification curves for 106 (red), 107 (cyan), 108 (green), and 0 (blue) copies of synthetic Chlamydia trachomatis 16S rDNA templates are depicted in panel a. Amplicon accumulation was measured as increase in fluorescence of the intercalating dye EvaGreen. Melting curve analysis of amplicons was performed using the “Tm calling” protocol in the LightCycler 96 software (panel b). This analysis allows identification and distinction of target-derived amplicons whose Tm peak is distinct from the melting temperature of non-specific amplicons. Color coding of the melting peaks is the same as that of the amplification curves. Cq of detecting different template copies is plotted as a bar graph in panel c. Standard curve analysis performed using the “Abs quant” protocol in the LightCycler 96 software is depicted in panel d. (PDF) [file pone.0201681.s003.pdf]

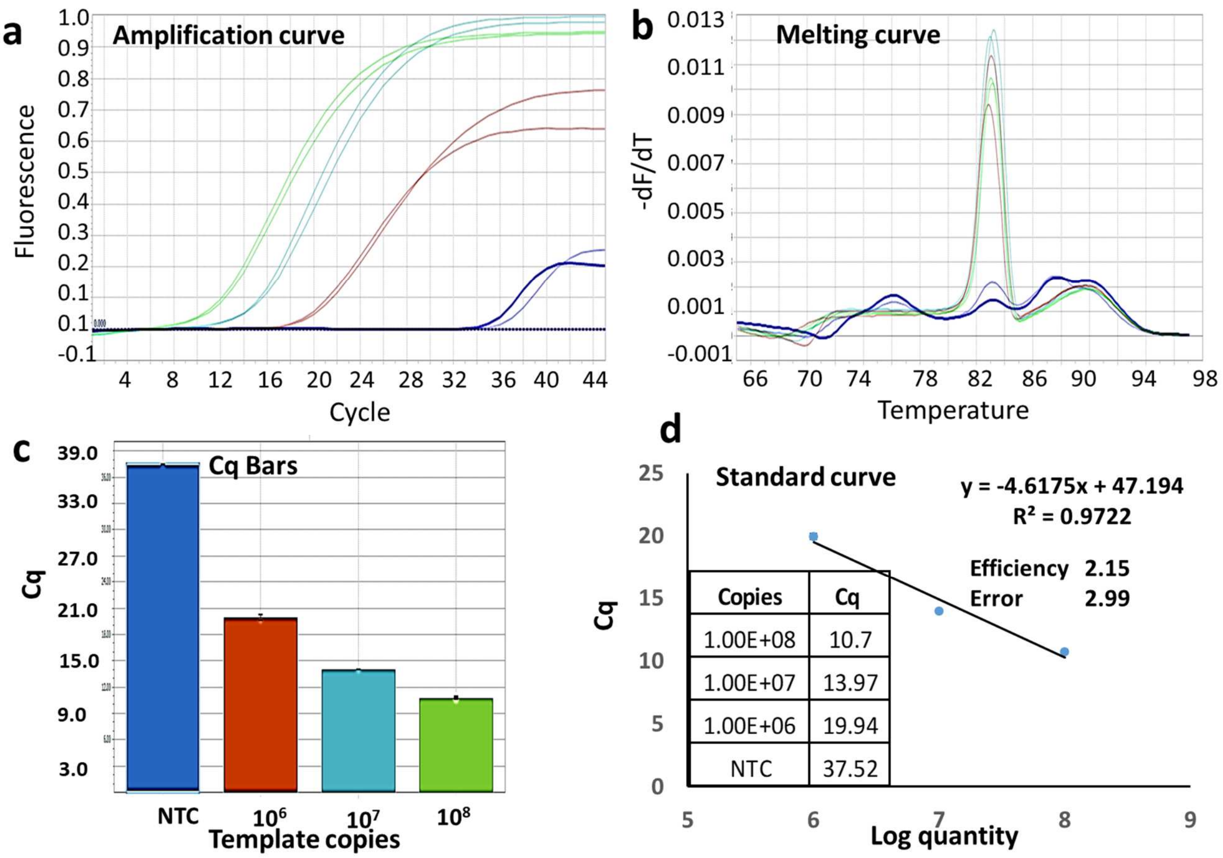

S3 Fig. qPCR analysis using lyophilized RTX Exo- expressing cellular reagents stored at room temperature for ~80 days. Real-time qPCR amplification curves for 10^6 (red), 10^7 (cyan), 10^8 (green), and 0 (blue) copies of synthetic *Chlamydia trachomatis* 16S rDNA templates are depicted in panel a. Amplicon accumulation was measured as increase in fluorescence of the intercalating dye EvaGreen. Melting curve analysis of amplicons was performed using the "Tm calling" protocol in the LightCycler 96 software (panel b). This analysis allows identification and distinction of target-derived amplicons whose Tm peak is distinct from the melting temperature of non-specific amplicons. Color coding of the melting peaks is the same as that of the amplification curves. Cq of detecting different template copies is plotted as a bar graph in panel c. Standard curve analysis performed using the "Abs quant" protocol in the LightCycler 96 software is depicted in panel d.
