# Supplementary material for: Cellular reagents for diagnostics and synthetic biology
Source: PLoS One. 2018 Aug 15;13(8):e0201681. doi: 10.1371/journal.pone.0201681 (PMC6093680; doi:10.1371/journal.pone.0201681)
Supplement: S4 Fig — BL21 E. coli expressing Taq DNA polymerase were lyophilized in either 1X PBS or in 1X PBS supplemented with 0.1M trehalose. After 3 days of storage at ambient temperature, the lyophilized cellular reagents were rehydrated in 30 μL water and half of the material was spread plated on Luria Bertani agar plates. Images of these plates were taken after overnight incubation at 37°C. Only bacteria that were lyophilized in the presence of trehalose retained viability. Cellular reagents lyophilized without trehalose do not remain viable. (PDF) [file pone.0201681.s004.pdf]

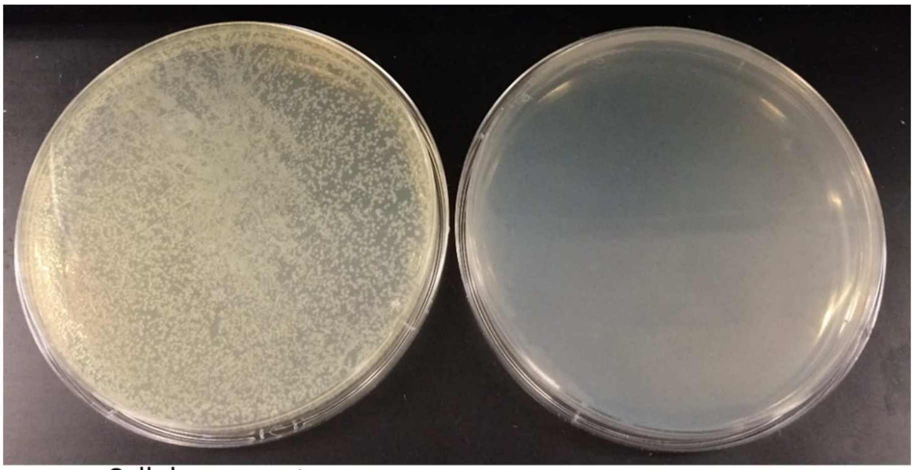

Cellular reagents lyophilized in 1X PBS containing 0.1M trehalose

Cellular reagents lyophilized in 1X PBS

S4 Fig. Assessment of bacterial viability in cellular reagents. BL21 *E. coli* expressing Taq DNA polymerase were lyophilized in either 1X PBS or in 1X PBS supplemented with 0.1M trehalose. After 3 days of storage at ambient temperature, the lyophilized cellular reagents were rehydrated in 30 µL water and half of the material was spread plated on Luria Bertani agar plates. Images of these plates were taken after overnight incubation at 37 °C. Only bacteria that were lyophilized in the presence of trehalose retained viability. Cellular reagents lyophilized without trehalose do not remain viable.
