# Supplementary material for: Cellular reagents for diagnostics and synthetic biology
Source: PLoS One. 2018 Aug 15;13(8):e0201681. doi: 10.1371/journal.pone.0201681 (PMC6093680; doi:10.1371/journal.pone.0201681)
Supplement: S5 Fig — BL21 E. coli cells overexpressing Taq DNA polymerase were washed in PBS and assessed for enzyme activity in three different conditions: fresh cells (FR), cells frozen at -80°C (FO), or lyophilized (L) cells. Cells (C) were tested isothermally by single step overlap extension assays at four different temperatures– 37°C, 42°C, 65°C, and 75°C. The PBS supernatants (S) leftover after pelleting fresh (SFR) or frozen (SFO) cells were also tested for polymerase activity. Overlap extension performed using pure (P) commercial Taq DNA polymerase served as the positive control. Reactions performed in the presence of oligonucleotide templates are labeled ‘Templates’. Negative controls lacking templates are denoted as ‘NTC’. All overlap extension products (indicated by ‘*’) were analyzed by agarose gel electrophoresis. Overlap extension template oligonucleotides (O; indicated with ‘#’) were analyzed as controls. (PDF) [file pone.0201681.s005.pdf]

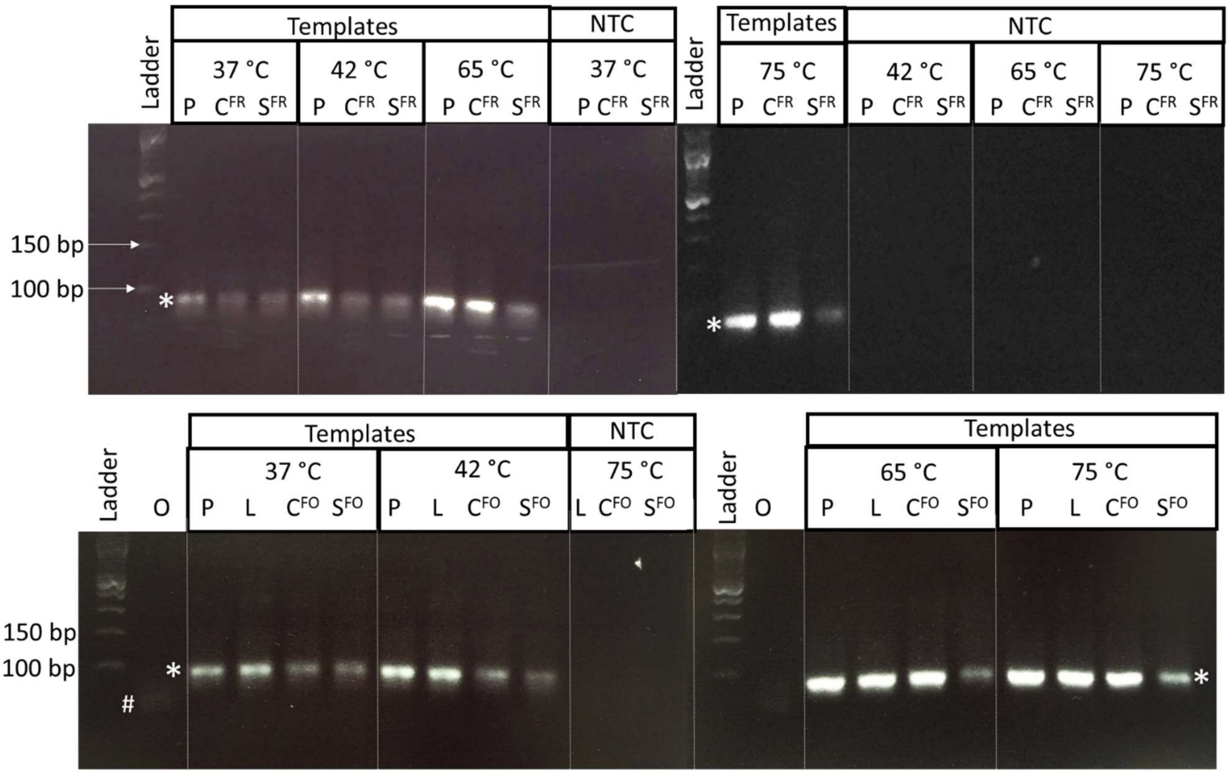

S5 Fig. Overlap extension assays to evaluate enzyme accessibility in cellular reagents. BL21 *E. coli* cells overexpressing Taq DNA polymerase were washed in PBS and assessed for enzyme activity in three different conditions: fresh cells (FR), cells frozen at -80 ℃ (FO), or lyophilized (L) cells. Cells (C) were tested isothermally by single step overlap extension assays at four different temperatures – 37 ℃, 42 ℃, 65 ℃, and 75 ℃. The PBS supernatant s (S) leftover after pelleting fresh (S^{FR}) or frozen (S^{FO}) cells were also tested for polymerase activity. Overlap extension performed using pure (P) commercial Taq DNA polymerase served as the positive control. Reactions performed in the presence of oligonucleotide templates are labeled ‘Templates’. Negative controls lacking templates are denoted as ‘NTC’. All overlap extension products (indicated by ‘*’) were analyzed by agarose gel electrophoresis. Overlap extension template oligonucleotides (O; indicated with ‘#’) were analyzed as controls.
