# Supplementary material for: Cellular reagents for diagnostics and synthetic biology
Source: PLoS One. 2018 Aug 15;13(8):e0201681. doi: 10.1371/journal.pone.0201681 (PMC6093680; doi:10.1371/journal.pone.0201681)
Supplement: S6 Fig — Freshly cultured E. coli cells overexpressing RTX DNA polymerase were washed and resuspended either in 1X PBS (a) or in water (b) prior to Gram staining and microscopic imaging under oil immersion and a 100X objective lens. Aliquots of these cells were also lyophilized and then rehydrated with water prior to microscopy. Cells lyophilized in 1X PBS are depicted in panel c while lyophilized cells examined after heat treatment are depicted in panels d (cells lyophilized in 1X PBS) and e (cells lyophilized in water). (PDF) [file pone.0201681.s006.pdf]

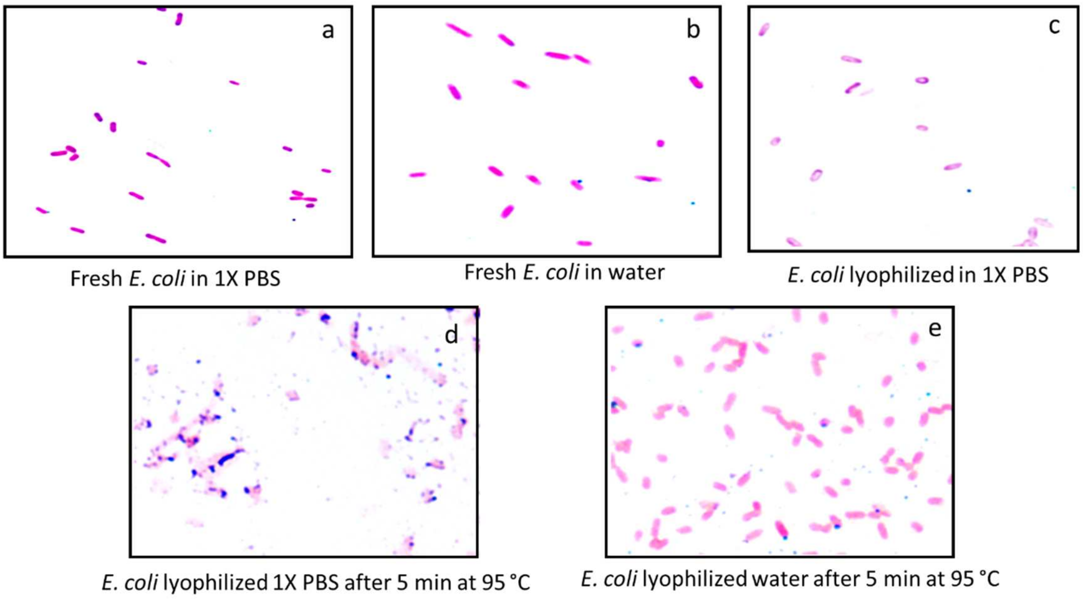

S6 Fig. Microscopic examination of cellular reagents. Freshly cultured *E. coli* cells overexpressing RTX DNA polymerase were washed and resuspended either in 1X PBS (a) or in water (b) prior to Gram staining and microscopic imaging under oil immersion and a 100X objective lens. Aliquots of these cells were also lyophilized and then rehydrated with water prior to microscopy. Cells lyophilized in 1X PBS are depicted in panel c while lyophilized cells examined after heat treatment are depicted in panels d (cells lyophilized in 1X PBS) and e (cells lyophilized in water).
