# Supplementary material for: Cellular reagents for diagnostics and synthetic biology
Source: PLoS One. 2018 Aug 15;13(8):e0201681. doi: 10.1371/journal.pone.0201681 (PMC6093680; doi:10.1371/journal.pone.0201681)
Supplement: S7 Fig — Taq DNA polymerase expressing cellular reagents stored with desiccant at 25°C, 37°C, or 42°C were tested for activity by using 2 x 10^7^ cells per reaction in endpoint PCR. Products were analyzed by gel electrophoresis and compared to PCR performed using 2.5 units of pure commercial Taq DNA polymerase. Activity of cellular reagents after 21 days of storage are depicted. (PDF) [file pone.0201681.s007.pdf]

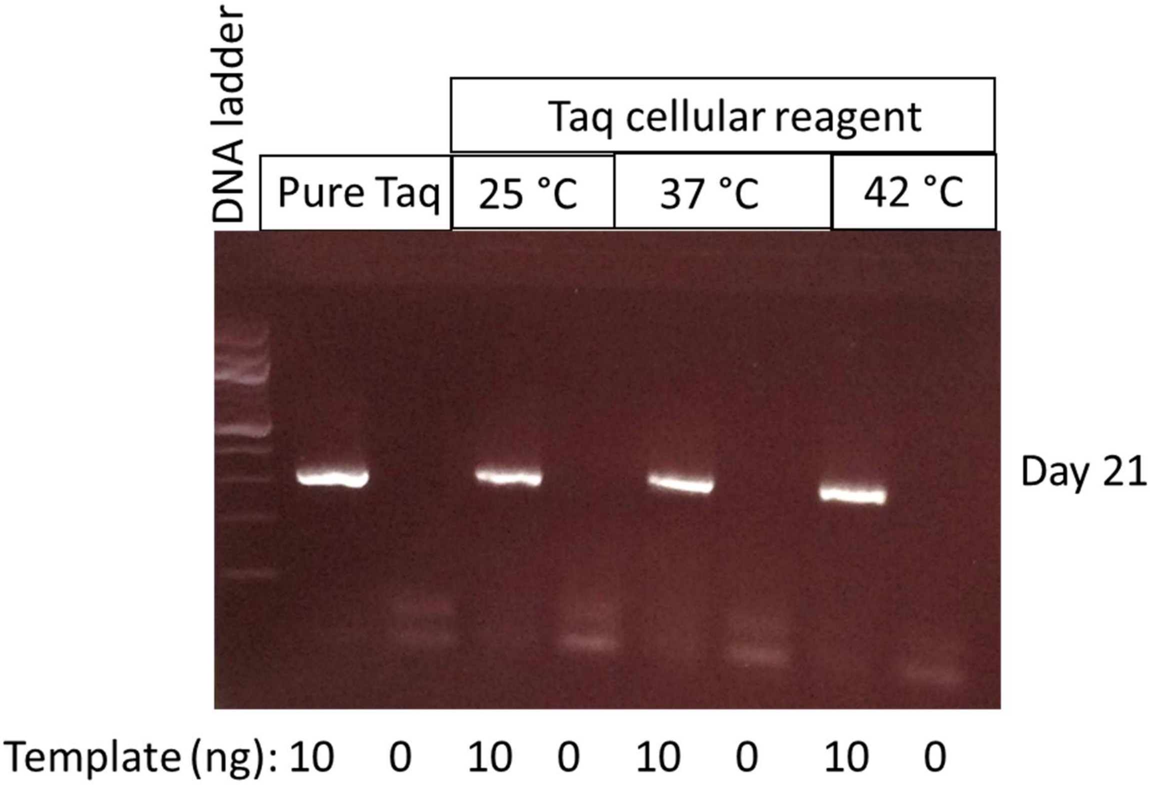

S7 Fig. Storage stability of Taq DNA polymerase cellular reagents at elevated temperatures. Taq DNA polymerase expressing cellular reagents stored with desiccant at 25 ℃, 3 7 ℃, or 42 ℃ were tested for activity by using 2 x 10^7 cells per reaction in endpoint PCR. Products were analyzed by gel electrophoresis and compared to PCR performed using 2.5 units of pure commercial Taq DNA polymerase. Activity of cellular reagents after 21 days of storage are depicted.
