# Supplementary material for: Cellular reagents for diagnostics and synthetic biology
Source: PLoS One. 2018 Aug 15;13(8):e0201681. doi: 10.1371/journal.pone.0201681 (PMC6093680; doi:10.1371/journal.pone.0201681)
Supplement: S8 Fig — A. Zika virus derived synthetic RNA template was analyzed by one-pot qRT-PCR using the Evoscript RNA Probes Master mix (Roche) according to the manufacturer’s instructions. Briefly, indicated RNA template copies were added to 1 X qRT-PCR master mix supplemented with 800 nM each of Zika 4481_F and Zika 4552c forward and reverse primers, and 200 nM of Zika 4507c-FAM TaqMan probe. PCR reactions were first incubated at 60°C for 30 min to allow reverse transcription. The reactions were then incubated at 95°C for 10 min prior to executing 45 cycles of 15 sec at 95°C and 30 sec at 55°C. Amplicon accumulation was measured as increase in TaqMan probe fluorescence. Amplification curves obtained using indicated copies of template RNA are depicted. These curves were generated using “Abs quant” analysis protocol in the LightCycler 96 software. B. Standard curve analysis of real-time amplification data shown in panel A. (PDF) [file pone.0201681.s008.pdf]

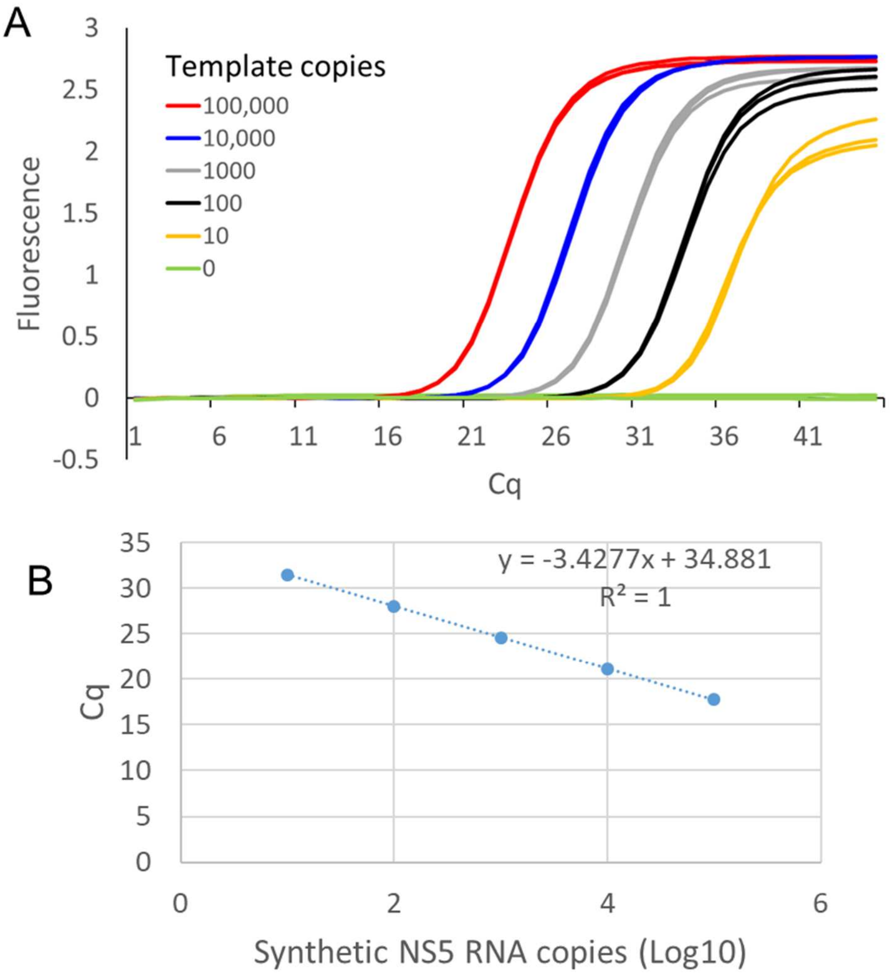

S8 Fig. Standard curve analysis of Zika virus RNA using commercial one-pot qRT-PCR master mix. A. Zika virus derived synthetic RNA template was analyzed by one-pot qRT-PCR using the Evoscript RNA Probes Master mix (Roche) according to the manufacturer's instructions. Briefly, indicated RNA template copies were added to 1 X qRT-PCR master mix supplemented with 800 nM each of Zika 4481_F and Zika 4552c forward and reverse primers, and 200 nM of Zika 4507c-FAM TaqMan probe. PCR reactions were first incubated at 60 ℃ for 30 min to allow reverse transcription. The reactions were then incubated at 95 ℃ for 10 min prior to executing 45 cycles of 15 sec at 95 ℃ and 30 sec at 55 ℃. Amplicon accum ulation was measured as increase in TaqMan probe fluorescence. Amplification curves obtained using indicated copies of template RNA are depicted. These curves were generated using "Abs quant" analysis protocol in the LightCycler 96 software. **B.** Standard curve analysis of real-time amplification data shown in panel A.
