# Supplementary material for: Cellular reagents for diagnostics and synthetic biology
Source: PLoS One. 2018 Aug 15;13(8):e0201681. doi: 10.1371/journal.pone.0201681 (PMC6093680; doi:10.1371/journal.pone.0201681)
Supplement: S10 Fig — Amplification efficiencies of lyophilized cellular reagents expressing Bst-LF DNA polymerase were tested in LAMP-OSD assays using indicated template copies. LAMP amplicon accumulation was measured in real-time using fluorogenic OSD probes. Cq values (time-to-signal) at different template copies determined using the “Abs quant” analysis protocol in the LightCycler 96 software are depicted. (PDF) [file pone.0201681.s010.pdf]

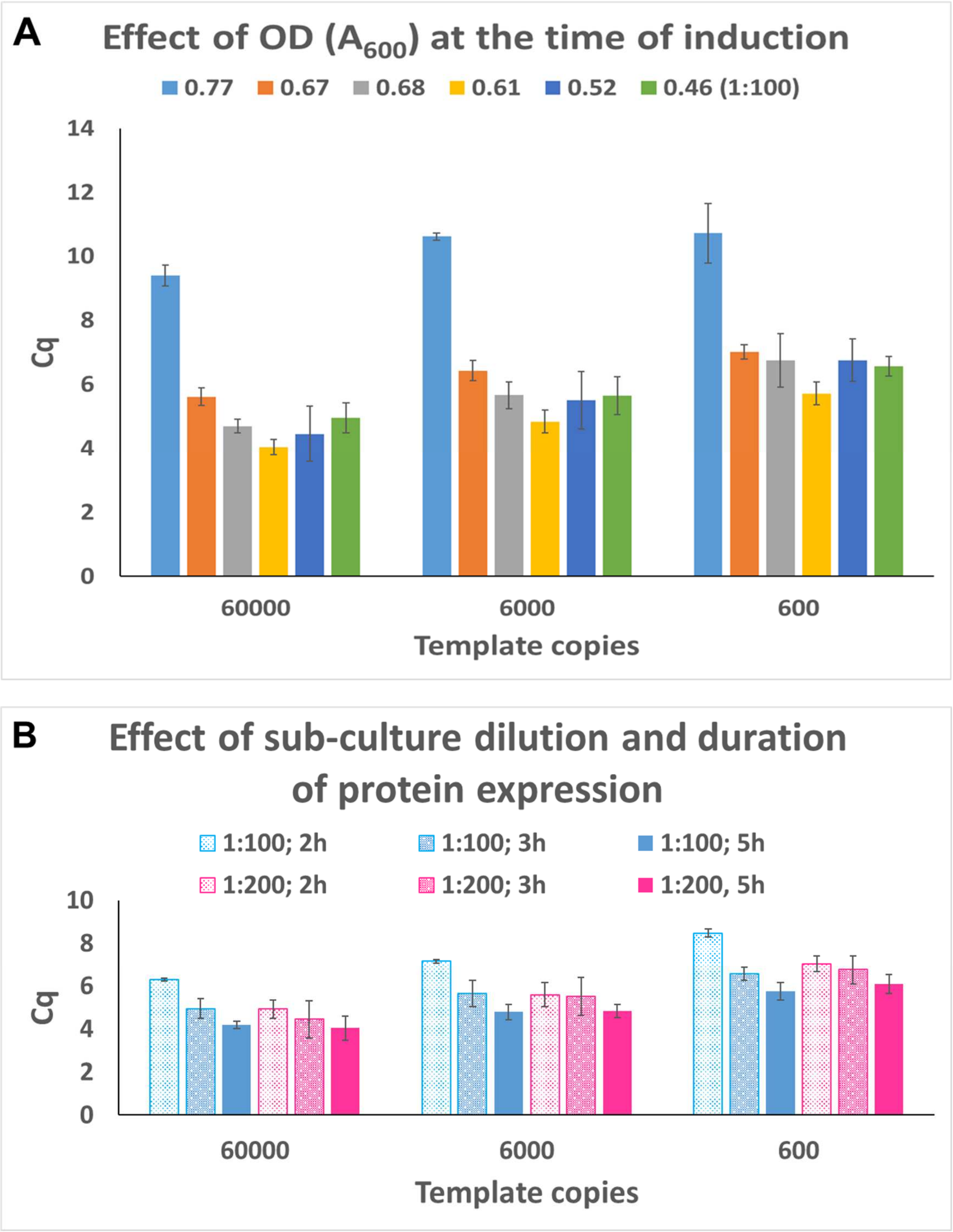

S10 Fig. Effect of culture conditions on performance of cellular reagents. Amplification efficiencies of lyophilized cellular reagents expressing Bst-LF DNA polymerase were tested in LAMP-OSD assays using indicated template copies. LAMP amplicon accumulation was measured in real-time using fluorogenic OSD probes. Cq values (time-to-signal) at different template copies determined using the “Abs quant” analysis protocol in the LightCycler 96 software are depicted.
