# Supplementary material for: Cellular reagents for diagnostics and synthetic biology
Source: PLoS One. 2018 Aug 15;13(8):e0201681. doi: 10.1371/journal.pone.0201681 (PMC6093680; doi:10.1371/journal.pone.0201681)
Supplement: S11 Fig — Amplification efficiencies of lyophilized cellular reagents expressing Bst-LF DNA polymerase were tested in LAMP-OSD assays using indicated template copies. LAMP amplicon accumulation was measured in real-time using fluorogenic OSD probes. Amplification curves obtained with either 60,000 (full traces labeled “(+)”) or 0 (dashed traces labeled “(-)”) copies of gapd templates are depicted. Amplification curves were generated using the “Abs quant” analysis protocol in the LightCycler 96 software. Bst-LF cellular reagents were either tested immediately after lyophilization (black traces labeled “Same day”) or after storage with desiccants for 21 days at 25°C (green traces labeled “25 C”), 37°C (pink traces labeled “37 C”), or 42°C (blue traces labeled “42 C”). (PDF) [file pone.0201681.s011.pdf]

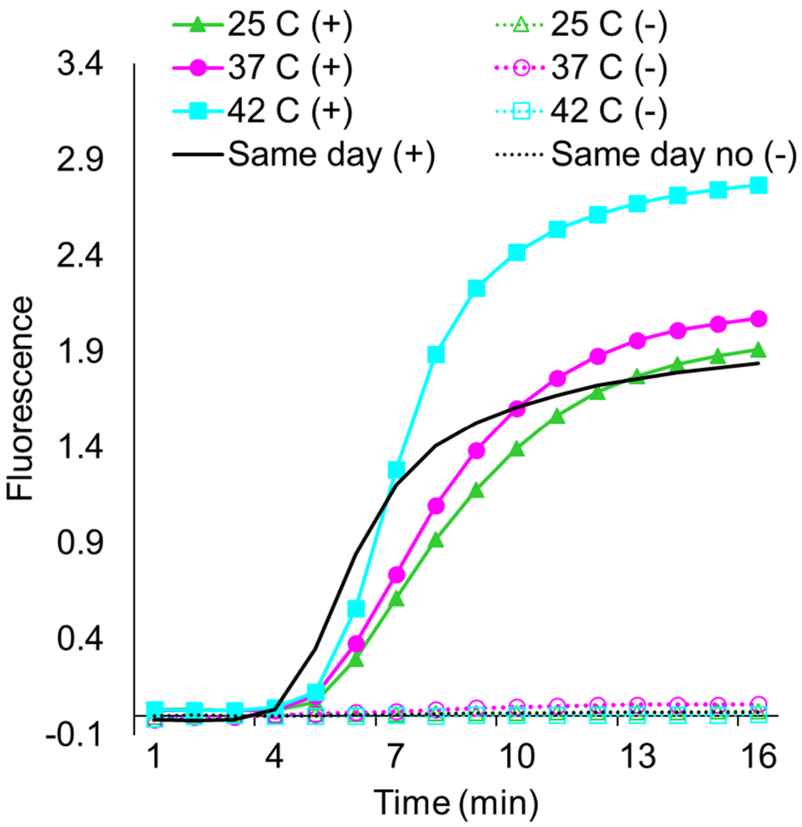


**S11 Fig. Storage stability of Bst-LF cellular reagents at elevated temperatures.** Amplification efficiencies of lyophilized cellular reagents expressing Bst-LF DNA polymerase were tested in LAMP-OSD assays using indicated template copies. LAMP amplicon accumulation was measured in real-time using fluorogenic OSD probes. Amplification curves obtained with either 60,000 (full traces labeled “(+)”) or 0 (dashed traces labeled “(-)”) copies of *gapd* templates are depicted. Amplification curves were generated using the “Abs quant” analysis protocol in the LightCycler 96 software. Bst-LF cellular reagents were either tested immediately after lyophilization (black traces labeled “Same day”) or after storage with desiccants for 21 days at 25 ℃ (green traces labeled “25 C”), 37 ℃ (pink traces labeled “37 C”), or 42 ℃ (blue traces labeled “42 C”).
