# Supplementary material for: Cellular reagents for diagnostics and synthetic biology
Source: PLoS One. 2018 Aug 15;13(8):e0201681. doi: 10.1371/journal.pone.0201681 (PMC6093680; doi:10.1371/journal.pone.0201681)
Supplement: S1 Table — (PDF) [file pone.0201681.s012.pdf]

S1 Table. Oligonucleotide and template sequences used in the study.

Name	Sequence	Use
CT-F	TAGTGGCGGAAGGGTTAG	*Chlamydia trachomatis* qPCR
CT-R	CGTCATAGCCTTGGTAGG	
Chlamydia trachomatis template	CGCCAAGCTTGGTACCGAGCTCGGATCCACTAGTAACGGCCGCCAGTGTGCTGGAA<br>TTCTAATACGACTCACTATAGGGCAATTGTTTAGTGGCGGAAGGGTTAGTAATGCATA<br>GATAATTTGTCCTTAACTTGGGAATAACGGTTGGAAACGGCCGCTAATACCGAATGT<br>GGCGATATTTGGGCATCCGAGTAACGTTAAAGAAGGGGATCTTAGGACCTTTCGGTT<br>AAGGGAGAGTCTATGTGATATCAGCTAGTTGGTGGGGTAAAGGCCTACCAAGGCTAT<br>GACGTCTAGGCGGATTGAGAGATTGGCCGCCAACACTGGGACTGAGACACTGCCCA<br>GACTCCTACGGGAGGCTGCAGTCGAGAATCTTTCGCAATGGACGGAAGTCTGACGA<br>AGCGACGCCGCGTGTGTGATGAAGGCTCTAGGGTTGTAAAGGAATTCTGCAGATAT<br>CCATCACACTGGCGGCCGCTCGAGCATGCATCTAGAGGGCCCAATT	
Zika-255-F	GGTAGATCCATTGTGGTCCCTTGCC	Zika Evagreen q(RT)PCR
Zika-256-R	CCACACCATGAGCATGTCCTCAGTAGTC	
Zika virus template	CTAGTAACGGCCGCCAGTGTGCTGGAATTCGGTAGATCCATTGTGGTCCCTTGCCG<br>CCACCAAGATGAATTGATTGGCCGAGCCCGTGTATCACCAGGGGCAGGATGGAGCA<br>TTCGGGAGACTGCCTGTCTAGCAAAATCATATGCACAGATGTGGCAGCTTCTTTACT<br>TCCACAGAAGAGACCTTCGACTGATGGCCAATGCTATTTGTTCGGCTGTGCCAGTTG<br>ACTGGGTACCAACCGGGAGAACCACCTGGTCAATCCACGGAAAGGGAGAATGGATG<br>ACTACTGAGGACATGCTCATGGTGTGGAATAGAGTGTGGATTGAGGAGGAATTCTGC<br>AGATATCCATCACACTGGCGGCCGCTCGAGC	
gapdLAMP.F3	GCCACCCAGAAGACTGTG	gapd LAMP-OSD
gapdLAMP.B3	TGGCAGGTTTTTCTAGACGG	
gapdLAMP.FIP	CGCCAGTAGAGGCAGGGATGAGGGAAACTGTGGCGTGAT	
gapdLAMP.BIP	GGTCATCCCTGAGCTGAACGGTCAGGTCCACCACTGACAC	
gapdLAMP.LR	TGTTCTGGAGAGCCCCGCGGCC	
gapdOSD.F	/56-FAM/CTCACTGGCATGGCCTTCCGTGTCCCCACTGCCAAC/3InvdT/	
gapdOSD.Q	GGACACGGAAGGCCATGCCAGTGAG/3IABkFQ/	
gapd template	CTAGTAACGGCCGCCAGTGTGCTGGAATTCCCACAGTCCATGCCATCACTGCCACC<br>CAGAAGACTGTGGATGGCCCCTCCGGGAAACTGTGGCGTGATGGCCGCGGGGCTC<br>TCCAGAACATCATCCCTGCCTCTACTGGCGCTGCCAAGGCTGTGGGCAAGGTCATC<br>CCTGAGCTGAACGGGAAGCTCACTGGCATGGCCTTCCGTGTCCCCACTGCCAACGT<br>GTCAGTGGTGGACCTGACCTGCCGTCTAGAAAAACCTGCCAAATATGATGACATCAA<br>GAAGGTGGTGAAGCAGGCGTCGGAGGGCCCCCTCAAGGGCATCCTGGGCTACACT<br>GAGCACCAGGTGGTCTCCTCTGACTTCAACAGCGACACCCACTCCTCCACCTTTGAC<br>GCTGGGGCTGGCATTGCCCTCAACGACCACTTTGTCAAGCTCATTTCCTGGAATTCT<br>GCAGATATCCATCACACTGGCGGCCGCTCGAGC	
Zika 4481_F	CTGTGGCATGAACCCAATAG	Zika TaqMan q(RT)PCR
Zika 4552c	ATCCCATAGAGCACCACTCC	
Zika 4507c-FAM	/56-FAM/CCACGCTCCAGCTGCAAAGG/3IABkFQ/	
Zika TaqMan template	GGGAC CATCTGTGGCATGAACCCAA TAGCCATACC CTTTGCAGCT GGAGCGTGGT<br>ACGTGTATGT GAAGACTGGAAAAAGGAGTG GTGCTCTATG GGATGTGCCT	
SB.pATetO.R	GCG CCC TTC GAT GTG ATG GTG ATG GTG ATG CGA TCC TCT G	Cellular PCR primers for pATetO 6XHis
SB.pATetO.F	TGATAATTGCCTCTGCCAAAATTCTGTCCTCAAGCGTTTTAGTTCG	
SB.Kan.ptet. F	GATCGCATCACCATCACCATCACATCGAAGGGCGC<br>GCTGAAAGCCAATTCTGATTAGAAAAACTC	Cellular PCR primers for kan^r insert for pATetO 6XHis
SB.Kan.ptet.R	GAGGACAGAATTTTGGCAGAGGCAATTATCA GATCCTTTGATCTCACGTTGTGTCTC	
SB.puc19FL.F	GAT CCC CGG GTA CCG AGC TCG AAT TCA CTG G	Cellular PCR primers for pUC19 fragments 1 and 2
SB.puc19.FL.R	CTC TAG AGT CGA CCT GCA GGC ATG CAA GCT TG	
SB. puc19sm.R	GAC AGT TAC CAA TGC TTA ATC AGT GAG GCA CC	
SB.puc19sm.F	GGTGCCTCACTGATTAAGCATTGGTAACTGTC	
SB.Kan.puc19.F	CAAGCTTGCATGCCTGCAGGTCGACTCTAGAGGCTGAAAGCCAATTCTGATTAGAAA<br>AACTC	Cellular PCR primers for kan^r insert for pUC19
SB.Kan.puc19.R	CCAGTGAATTCGAGCTCGGTACCCGGGGATC<br>GATCCTTTGATCTCACGTTGTGTCTC	
OE.FWD	TAATACGACTCACTATAGGGTGGTTTCTGGGGTGACCGGGTTGATTCTCAGCCCTTC<br>GCA	Overlap extension assay
OE.REV	AGGGGTTGGTTGGATGAATATAGGGGATTGCGAAGGGCTGAGAATCAACCCGGTCA<br>CCCC	

pCR2.1.FluB template	CTAGTAACGGCCGCCAGTGTGCTGGAATTCTAATACGACTCACTATAGGGATGTCGC<br>TGTTTGGAGACACAATTGCCTACTTGCTTTCATTAACAGAAGATGGAGAAGGCAAAG<br>CAGAACTAGCAGAAAAGTTACACTGTTGGTTTGGTGGGAAAGAATTTGACCTAGACT<br>CAGCCTTGGAATGGATAAAAAACAAAAGATGCTTAACTGATATACAAAAAGCACTAAT<br>TGGTGCCTCTATATGCTTTTTAAAACCCAAAGACCAGGAAAGAAAAAGAAGATTCATC<br>ACAGAATTCTGCAGATATCCATCACACTGGCGGCCGCTCGAGC	Taq DNA polymerase endpoint PCR
pCR.FWD	CTAGTAACGGCCGCCAGTGTGCTGGAATTC	
pCR.REV	CCGCCAGTGTGATGGATATCTGCAGAATTC	
