# Supplementary material for: Cellular reagents for diagnostics and synthetic biology
Source: PLoS One. 2018 Aug 15;13(8):e0201681. doi: 10.1371/journal.pone.0201681 (PMC6093680; doi:10.1371/journal.pone.0201681)
Supplement: S2 Table — (PDF) [file pone.0201681.s013.pdf]

S2 Table. Cellular reagent Gibson assembly with or without heat treatment of cellular reagents.

Gibson assembly type	Number of colonies
**Gel purified pCR2.1 linearized vector + *Aedes albopictus* S7 gBlock**	
Cellular reagent Gibson with heat-treated Taq DNA polymerase and Taq DNA Ligase	111 [a]
Cellular reagent Gibson with no heat treatment of cellular reagents	12
Gibson with pure enzymes (positive control)	183
Assembly with no enzymes (negative control)	1
**Gel purified pCR2.1 linearized vector + *E. coli yaiO* gBlock**	
Cellular reagent Gibson with heat-treated Taq DNA polymerase and Taq DNA Ligase	173
Gibson with pure enzymes (positive control)	360
Assembly with no enzymes (negative control)	1

[a]All tested positive control clones had the correct sequence. 50% of the cellular reagent Gibson assembled plasmids had the correct sequence. Remaining recovered colonies contained re-circularized vector.
