# Supplementary material for: Cellular reagents for diagnostics and synthetic biology
Source: PLoS One. 2018 Aug 15;13(8):e0201681. doi: 10.1371/journal.pone.0201681 (PMC6093680; doi:10.1371/journal.pone.0201681)

Cellular Reagents for Diagnostics and Synthetic Biology Statistical Supplement

Sanchita Bhadra, Arti Pothukuchy, Raghav Shroff, Austin Cole, Michelle Byrom, Jared W. Ellefson, Jimmy D. Gollihar, and Andrew D. Ellington

Summary: Cellular reagents significantly effect the expected CQ value of an amplification reaction, but their effect is small. In both cases tested the expected CQ value of a reaction involving cellular reagents is about 2 units larger than the expected CQ value of a reaction involving purified reagents.

The authors are interested in the effect of reagent type, cellular or purified, on expected cq values. The data used for this analysis has four attributes; a template copy count (copies), a CQ value for the reaction (cq), the cellular or purified reagent used (rgnt), and the reaction conditions (rxn). Data involving no cq readout or zero templates were omitted from analysis to enable linear regression. The data is formatted as depicted here:

```
##    copies   cq rgnt                rxn log_copies
## 1   6e+06 2.81    P LAMP-OSD (BST-2.0)   15.60727
## 2   6e+06 2.35    P LAMP-OSD (BST-2.0)   15.60727
## 3   6e+06 2.69    P LAMP-OSD (BST-2.0)   15.60727
## 4   6e+06 2.73    P LAMP-OSD (BST-2.0)   15.60727
## 5   6e+06 7.30    P  LAMP-OSD (BST-LF)   15.60727
## 6   6e+06 6.75    C  LAMP-OSD (BST-LF)   15.60727
```

Since LAMP and PCR based assays amplify templates with two distinct mechanisms, they are analyzed in separate models. First, the effect of reagent type on PCR like reactions is modeled and tested with an anova.

```
# The model ommitting reagent type #
lm.reduced.pcr = lm(cq ~ log_copies + rxn, data = Cell_rgnt_pcr)
# The model including reagent type #
lm.full.pcr = lm(cq ~ log_copies + rxn + rgnt, data = Cell_rgnt_pcr)
```

Diagnostic Plots for the Model Without Reagent Type

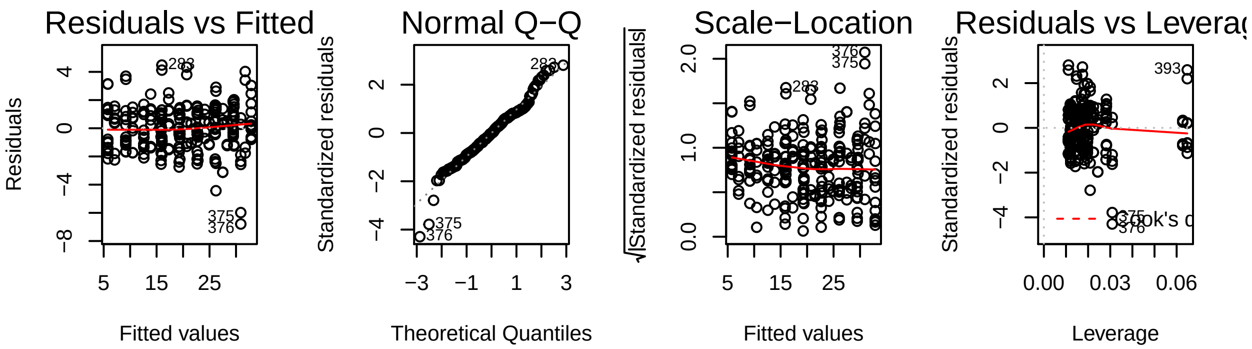

Diagnostic Plots for the Model With Reagent Type

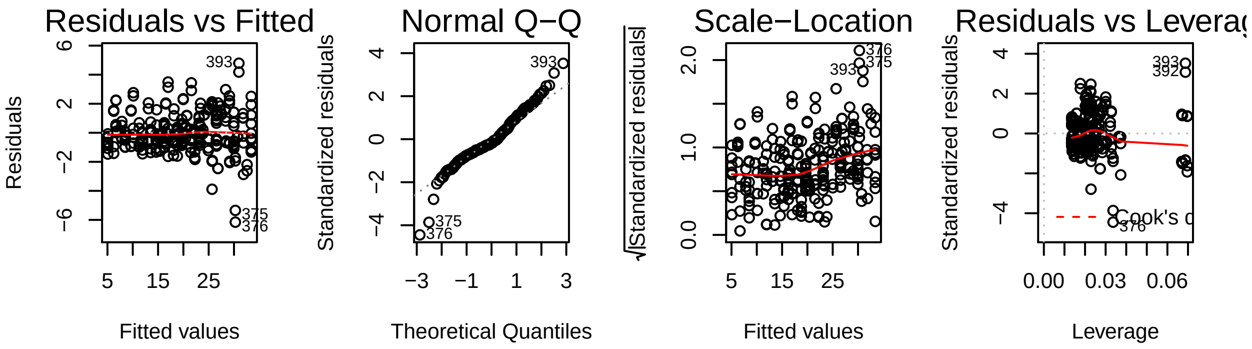

Since both models satisfy regression assumptions it is safe to perform an anova test. A test of two models omitting any interaction terms with CQ data from PCR like reactions suggests that the reagent type has a significant effect on the CQ.

```
## Analysis of Variance Table
##
## Model 1: cq ~ log_copies + rxn + rgnt
## Model 2: cq ~ log_copies + rxn
##   Res.Df    RSS Df Sum of Sq      F    Pr(>F)
## 1    243 481.65
## 2    244 629.65 -1   -147.99 74.665 7.576e-16 ***
## ---
## Signif. codes:  0 '***' 0.001 '**' 0.01 '*' 0.05 '.' 0.1 ' ' 1
```

Despite its strong statistical significance, the effect of cellular reagents on CQ value is small. It is expected that a PCR like reaction using cellular reagents will be roughly 1.6 CQ units larger than a reaction using purified reagents.

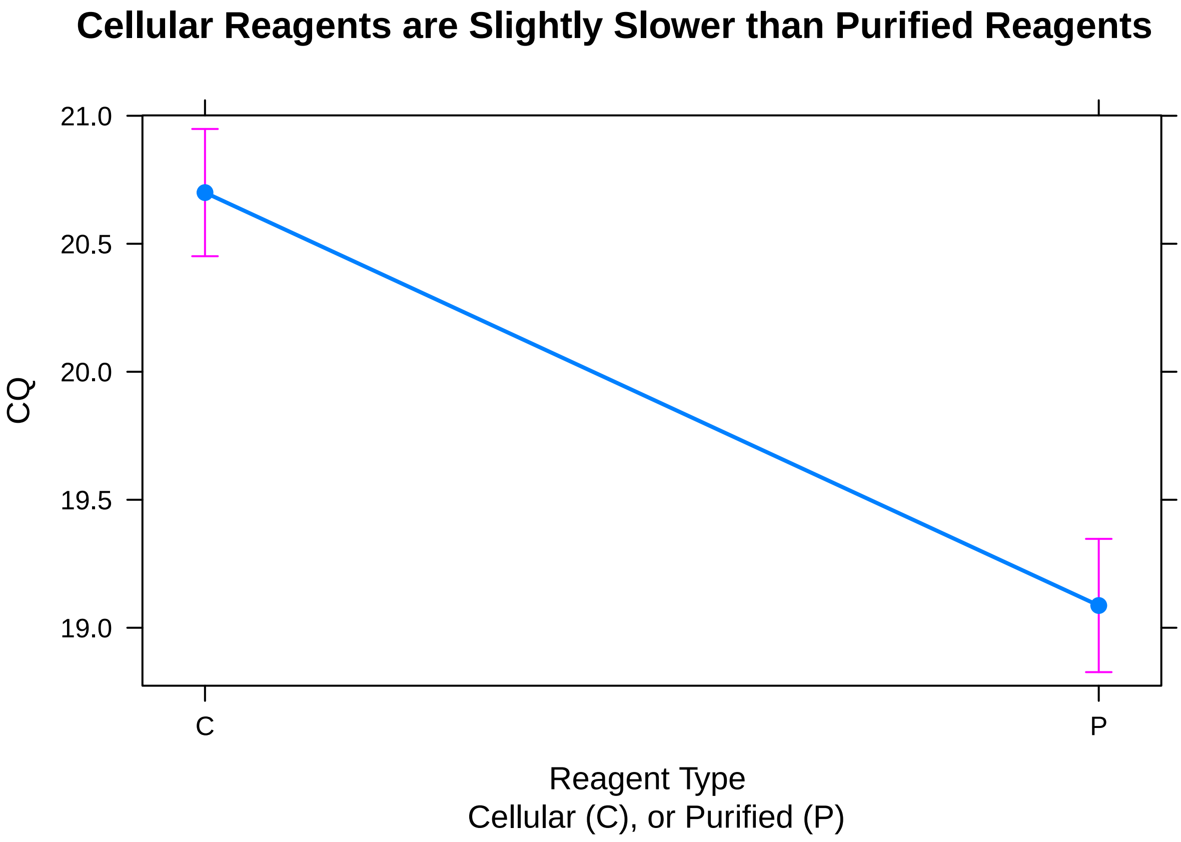

Experiments for LAMP reactions were not balanced–they involved cellular reagents for BST LF but not for BST 2.0. Since the comparison of interest is between cellular reagents and their market competition, the model will be built using experiments involving cellular BST LF and purified BST 2.0.

Here a model of cq value explained by 1) cellular BST LF or purified BST 2.0 and 2) template copy number will be tested with a type II anova.

```
# The model of CQ values as explained by template copy and reagent type #
lm.lamp = lm(cq ~ (log_copies + rgnt), data = Cell_rgnt_lamp_trm)
```

Diagnostic Plots for the Model with Interactions Between Predictors

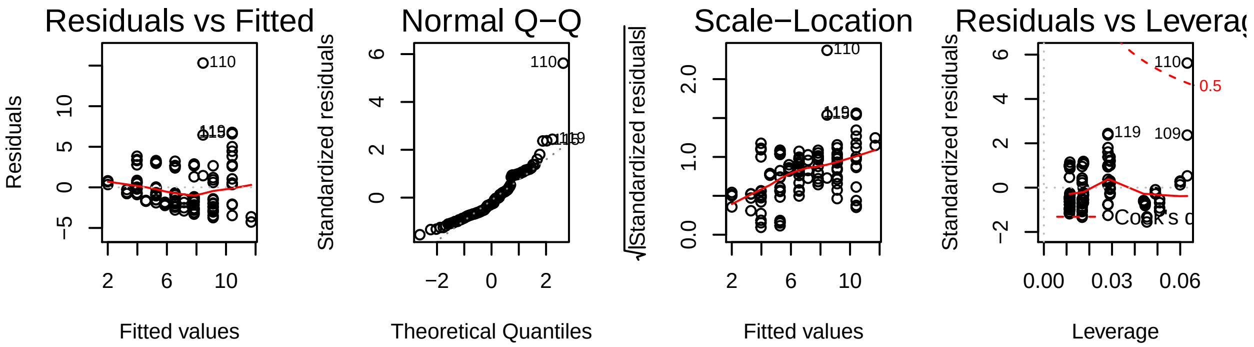

The model satisfies regression assumptions despite the outlier at index 110, so it is safe to perform an anova test. A type II anova detecting a significant effect of reagent type on CQ value is shown below.

```
## Anova Table (Type II tests)
```

```
##
## Response: cq
##             Sum Sq  Df F value    Pr(>F)
## log_copies 575.63   1 72.6115 7.446e-14 ***
## rgnt        71.83   1  9.0608  0.003216 **
## Residuals  903.74 114
## ---
## Signif. codes:  0 '***' 0.001 '**' 0.01 '*' 0.05 '.' 0.1 ' ' 1
```

Similar to PCR based reactions, cellular reagents have a significant effect on CQ value, but the size of their effect is small. The CQ value of a reaction performed with cellular BST LF is expected to be ~2 units larger than a reaction performed with purified LF.

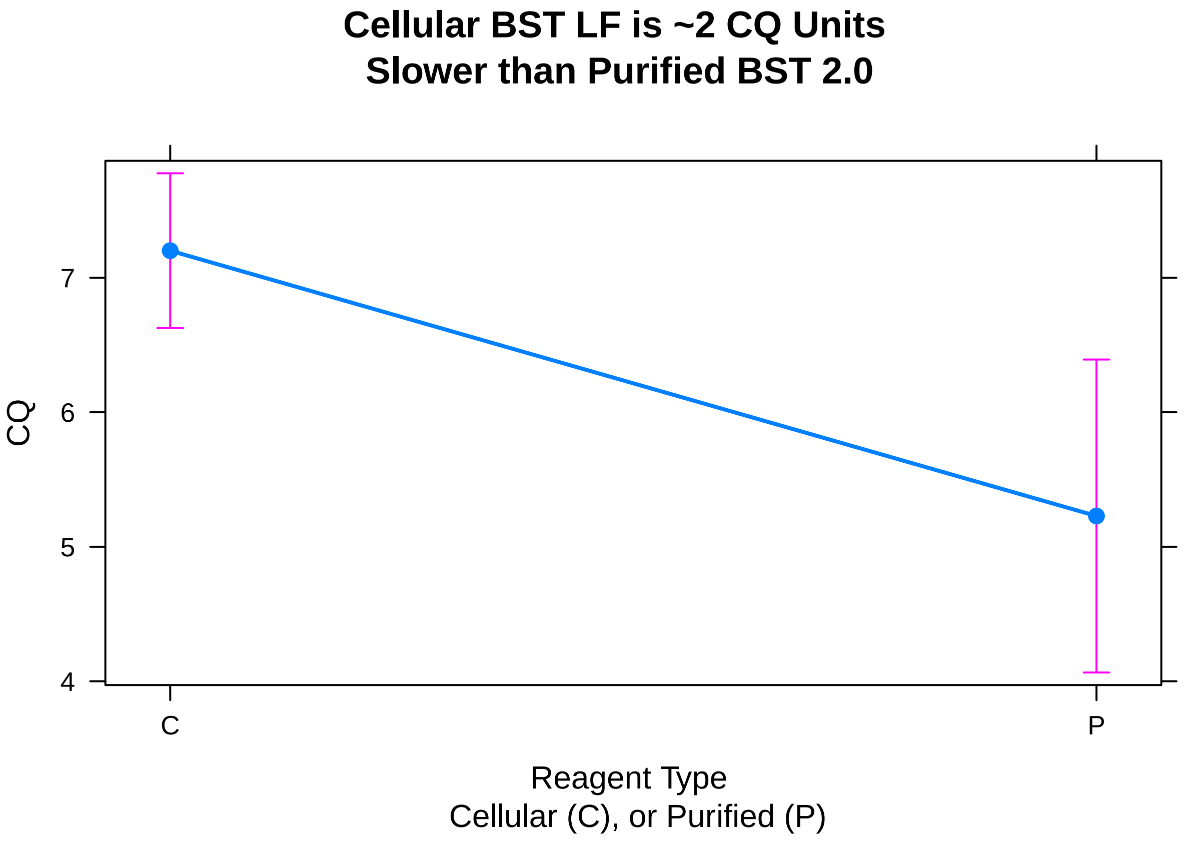

Supplement: S1 Statistical Supplement — (PDF) [file pone.0201681.s014.pdf]
